# Mass Spectrometric Analysis of TRPM6 and TRPM7 Phosphorylation Reveals Regulatory Mechanisms of the Channel-Kinases

**DOI:** 10.1038/srep42739

**Published:** 2017-02-21

**Authors:** Na Cai, Zhiyong Bai, Vikas Nanda, Loren W. Runnels

**Affiliations:** 1 Rutgers-Robert Wood Johnson Medical School, Dept. of Pharmacology, Piscataway, 08854, USA; 2 Rutgers-Center for Advanced Biotechnology and Medicine and Robert Wood Johnson Medical School, Dept. of Biochemistry and Molecular Biology, Piscataway, 08854, USA

## Abstract

TRPM7 and TRPM6 were the first identified bifunctional channels to contain their own kinase domains, but how these channel-kinases are regulated is poorly understood. Previous studies identified numerous phosphorylation sites on TRPM7, but very little is known about TRPM6 phosphorylation or sites on TRPM7 transphosphorylated by TRPM6. Our mass spectrometric analysis of homomeric and heteromeric TRPM7 and TRPM6 channels identified phosphorylation sites on both proteins, as well as several prominent sites on TRPM7 that are commonly modified through autophosphorylation and transphosphorylation by TRPM6. We conducted a series of amino acid substitution analyses and identified S1777, in TRPM7’s catalytic domain, and S1565, in TRPM7’s exchange domain that mediates kinase dimerization, as potential regulatory sites. The phosphomimetic S1777D substitution disrupted catalytic activity, most likely by causing an electrostatic perturbation at the active site. The S1565D phosphomimetic substitution also inactivated the kinase but did so without interfering with kinase dimerization. Molecular modeling indicates that phosphorylation of S1565 is predicted to structurally affect TRPM7’s functionally conserved N/D loop, which is thought to influence the access of substrate to the active site pocket. We propose that phosphorylation of S1565 within the exchange domain functions as a regulatory switch to control TRPM7 catalytic activity.

TRPM7 and its close homologue TRPM6 are bifunctional proteins consisting of a cation-permeating channel with a COOH-terminal functional kinase domain. TRPM6 was first linked to vertebrate Mg^2+^ homeostasis when identified as the ion channel mutated in the autosomal recessive disorder hypomagnesemia with secondary hypocalcemia^1^,^2^,^3^. TRPM7 was later shown also to play a role in whole-body magnesium homeostasis^4^,^5^. Unlike TRPM6, which is more selectively expressed in kidney and the colon, the ubiquitously expressed TRPM7 is implicated in many cellular functions, ranging from control of cell proliferation, cellular magnesium homeostasis, to cell adhesion and cell migration^6^,^7^,^8^. In addition, TRPM7 is critical for many developmental processes from the early embryo until later during organogenesis^9^,^10^,^11^.

The channel-kinases are also members of the atypical alpha-kinase family, so named after initial members of the family *Dictyostelium* myosin heavy chain kinase A, B, C (MHCK A, B, C), which primarily phosphorylate their substrates within alpha helical domains rather than within flexible loops and turns, as generally done by conventional protein kinases^12^. Following the identification of eukaryotic elongation factor-2 kinase (eEF2K), the membership of the alpha-kinase family quickly expanded across various genomes, including the two sister channel-kinases TRPM6 and TRPM7^13^. Due to TRPM6 and TRPM7’s unique status as the first identified channel-kinase fusion proteins, the functional interrelationship between the channel and kinase has been extensively investigated. The catalytic activity of TRPM7’s kinase is not required for channel gating, but the kinase itself appears to play a regulatory role in conferring the sensitivity of the channel activity to Mg^2+^ nucleotides^14^. Structural analysis of TRPM7 kinase reveals that despite little sequence similarity to conventional protein kinases, the tertiary structure of the kinase resembles the structure of conventional protein kinases like protein kinase A with some exceptions^15^,^16^. The kinase domain of TRPM7 assembles into functional dimers through the exchange of a short stretch of amino acids NH_2_-terminal to the core catalytic domain^17^. This exchange segment is highly conserved in both TRPM7 and TRPM6 and is required for kinase dimerization and the catalytic activity of both kinases^17^,^18^. Subsequent investigations into TRPM7 kinase functions have uncovered a number of *in vitro* substrates, including non-muscle myosin heavy chain IIA, annexin I, phospholipase C gamma-2, and histones, suggesting that the TRPM7 kinase may be impacting a diverse range of physiological activities^19^,^20^,^21^,^22^,^23^.

Unlike other members of the alpha-kinase family and conventional protein kinases, TRPM7 has been shown to undergo extensive autophosphorylation within a serine/threonine-rich region proximal to the exchange domain of the kinase and the kinase’s catalytic core^24^,^25^. Studies of other members of the alpha-kinases family such as eEF2K and *Dictyostelium* MHCKs identified important autophosphorylation sites outside the catalytic core that regulate kinase activity^26^,^27^; however, comparable regulatory phosphorylation sites have not been determined for TRPM7 or TRPM6. In addition, it was shown previously that autophosphorylation of TRPM7 does not appear to be a prerequisite for the kinase’s catalytic activity^24^. Instead, extensive phosphorylation of the serine/threonine-rich segment adjacent to the catalytic core was shown to facilitate phosphorylation of the substrate myosin IIA through enhanced substrate binding^24^. Interestingly, it was reported that in a heteromeric TRPM6/7 complex, TRPM7 is transphosphorylated by TRPM6^28^,^29^. TRPM7 has been shown to facilitate trafficking of TRPM6 to the plasma membrane and together they constitute a novel channel conductance when heterologously expressed^4^,^30^. TRPM7 intracellular trafficking is affected by TRPM6 kinase activity, but the mechanism by which this occurs is not clear^29^. Here we report our findings from proteomic and biochemical approaches to identify phosphorylated residues on TRPM7 and TRPM6 as a first step towards investigating the impact that phosphorylation has on channel-kinase function and regulation. Our results point to a critical role for phosphorylation in controlling the catalytic activity of the channel-kinases.

## Results

### Identification of Phosphorylation Sites on TRPM7 and TRPM6

While numerous studies have focused on regulation of the channel activity of TRPM7 and TRPM6, by comparison the functional role of these channels’ kinase domains or how they are regulated is poorly understood. In previous studies, a high number of phosphorylation sites were identified on TRPM7, either by performing mass spectrometry (MS) analysis of an ATP-stimulated COOH-terminal fragment of TRPM7 or of full length TRPM7 transiently expressed and purified from human embryonic kidney cells^24^,^25^. As technological advances have improved the sensitivity for identifying phosphorylation sites on proteins, we were motivated to discover sites that may have been missed in previous analyses of TRPM7, as well as to identify phosphorylation sites on TRPM6, whose phosphorylation has not yet been systematically investigated.

**Figure 1:**
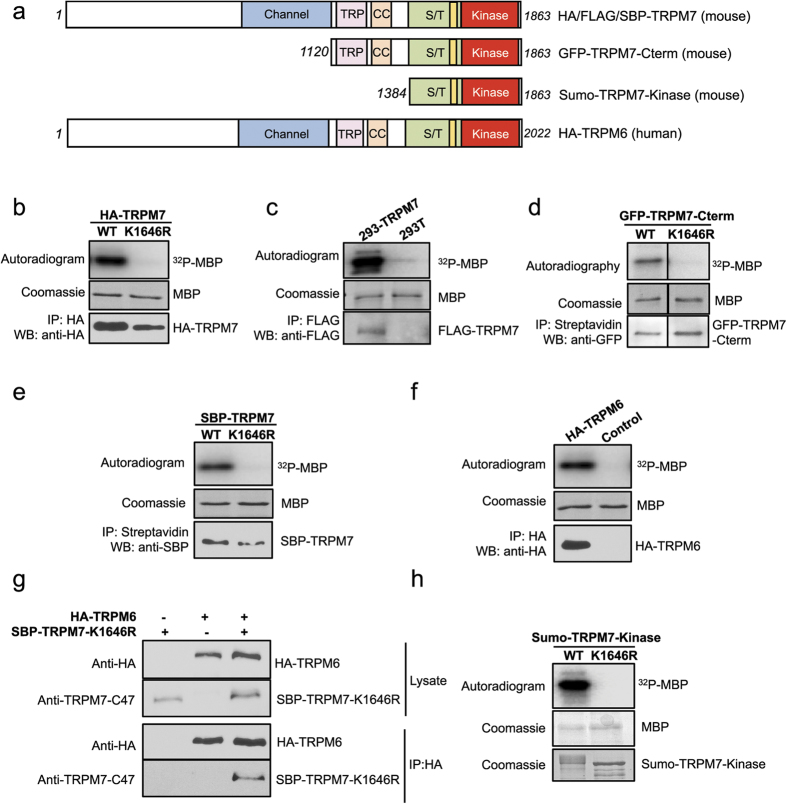
TRPM7 and TRPM6 constructs employed for phosphorylation analysis. (**a**) Schematic diagrams showing the domain organization of TRPM7 and TRPM6. Between the ion channel (blue) and the functional alpha-kinase domain (red) there reside a conserved TRP box (purple), a coiled-coil domain (orange), a serine/threonine-rich domain (green), and an exchange-segment of the kinase (yellow). Two additional TRPM7 constructs were generated: a GFP-tagged mouse TRPM7 C-terminus protein (a.a. 1120–1863) (GFP-TRPM7-Cterm) and a Sumo-tagged mouse TRPM7 fragment containing the S/T-rich and kinase domains (a.a. 1384–1683) (Sumo-TRPM7-Kinase). TRP = TRP box, CC = coiled-coil domain, S/T = serine/threonine-rich domain. (**b**–**f**) Various TRPM7 and TRPM6 proteins were purified from mammalian cells as described in the Material and Methods. The kinase activities of purified proteins were assessed *in vitro* with kinase assays using myelin basic protein (MBP) as a substrate, and the assays were performed at 30 °C for 20 min. The proteins were resolved by SDS-PAGE and Coomassie blue staining. ^32^P incorporation into MBP was detected by autoradiography. The presence of each kinase in the sample was verified by western blotting. (**g**) Co-immunoprecipitation assay confirms the heteromeric TRPM6/7 complex formation. A kinase-inactive SBP-TRPM7-K1646R was co-expressed with HA-TRPM6 in HEK-293T cells and pulled down by HA-agarose. Proteins in the lysate and the immunoprecipitated samples were resolved by SDS-PAGE and analyzed by western blotting. (**h**) Sumo-TRPM7-Kinase WT and K1646R were purified from *E. Coli* as described in the Materials and Methods. Their catalytic activities were tested *in vitro* in a kinase assay using MBP as a substrate and the assays were performed at 30 °C for 2 min.

**Figure 2:**
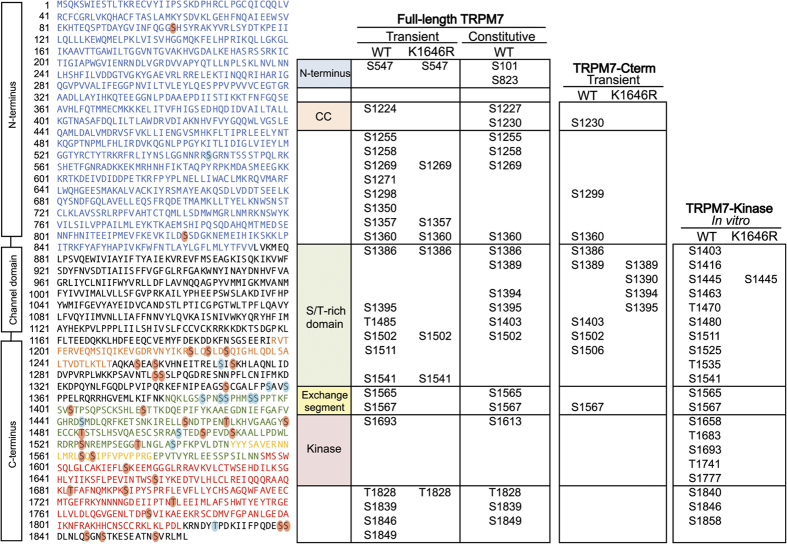
Identification of phosphorylated residues on TRPM7 by LC-MS/MS. The diagram on the left depicts the amino acid sequence of mouse TRPM7 (UniProtKB: Q923J1). The cytosolic N-terminus, the coiled-coil domain, the S/T-rich domain, the exchange segment, and the alpha-kinase domain are colored in blue, orange, green, yellow and red respectively. Phosphorylated residues identified exclusively in the TRPM7-WT samples are highlighted with red circles. Sites also identified in the K1646R samples are shown in blue circles. The chart on the right summarizes identified phosphorylation residues from various TRPM7 constructs used in the study.

We employed liquid chromatography tandem mass spectrometry (LC-MS/MS) to identify residues phosphorylated on TRPM7 and TRPM6, using different constructs both *in vivo* and *in vitro* (Fig. 1a). We first purified wild-type (WT) HA-tagged mouse TRPM7 (HA-TRPM7) or a kinase-inactive mutant (HA-TRPM7-K1646R) transiently expressed in HEK-293T cells. The kinase activities of purified proteins were tested *in vitro* via kinase assays using myelin basic protein (MBP) as a substrate (Fig. 1b). Unlike what was previously reported for purified COOH-terminal fragment of TRPM7, where “massive” autophosphorylation was observed^24^, our mass spectrometric analysis identified more selective *in vivo* phosphorylation of full-length TRPM7 in the absence of ATP stimulation (Fig. 2). A total of twenty-three residues were identified on the full-length HA-TRPM7, among which fifteen were exclusive to the WT kinase and were not identified in the kinase-inactive mutant. By comparing the relative abundance of each of the phosphopeptides on the MS/MS spectra, we identified S1567 as the major phosphorylation site on the overexpressed TRPM7 (Supplementary Table 1). We previously showed that overexpressing HA-TRPM7 in HEK-293T cells could lead to successful localization of the channel to cell surface, but also leave behind a lot of overexpressed proteins in the endoplasmic reticulum (ER)^7^,^11^. We were, therefore, concerned that phosphorylation events that occur at the plasma membrane could potentially be underrepresented in our sample. To obtain a more physiologically representative analysis of the phosphorylation state of TRPM7 *in vivo*, we employed a tetracycline-inducible HEK-293-TRPM7 cell line created by Nadler and colleagues^31^. In this cell line constitutively expressed FLAG-tagged mouse TRPM7 (FLAG-TRPM7) can be detected in the absence of tetracycline (Fig. 1c), with the added benefit that the cells are not stressed and are in equilibrium, with a modestly high expression of TRPM7^31^. LC-MS/MS analysis identified twenty phosphorylation sites on the constitutively expressed TRPM7 (Fig. 2) and under these conditions residue S1360 was recognized as the most frequently phosphorylated site (Supplementary Table 2). Recently, TRPM7 has been reported to be subject to proteolytic cleavage at its COOH-terminus, releasing fragments containing the catalytically active kinase^23^,^32^. We thus undertook an analysis of the phosphorylation pattern of the COOH-terminal fragment of TRPM7 (Fig. 1a,d). We employed a construct where the COOH-terminal fragment of mouse TRPM7 (a.a. 1120–1863) was fused to a multifunctional GFP-tag containing a streptavidin binding protein motif (GFP-TRPM7-Cterm). When GFP-TRPM7-Cterm is transiently expressed in HEK-293T cells, it is localized to the cytoplasm^11^. LC-MS/MS analysis of purified GFP-TRPM7-Cterm WT and GFP-TRPM7-Cterm-K1646R revealed a total of twelve phosphorylation sites, with eight of them exclusive to the WT kinase (Fig. 2 and Supplementary Table 3).

**Figure 3:**
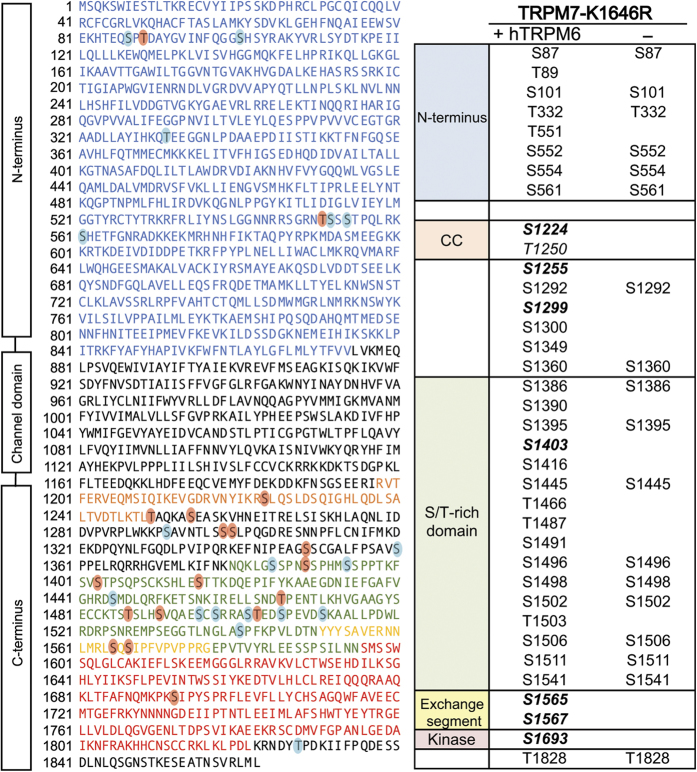
Identification of TRPM7 transphosphorylation sites by TRPM6. The diagram on the left depicts the amino acid sequence of mouse TRPM7 (UniProtKB: Q923J1). The cytosolic N-terminus, the coiled-coil domain, the S/T-rich domain, the exchange segment, and the alpha-kinase domain are colored in blue, orange, green, yellow and red respectively. Phosphorylated residues introduced by TRPM6 on TRPM7 are displayed by red circles and residues identified in the control kinase-inactive TRPM7 are displayed as blue circles. The chart on the right summarizes all identified phosphorylation residues from the analysis. Bold italic represents residues on TRPM7 that are both autophosphorylated and transphosphorylated by TRPM6.

**Figure 4:**
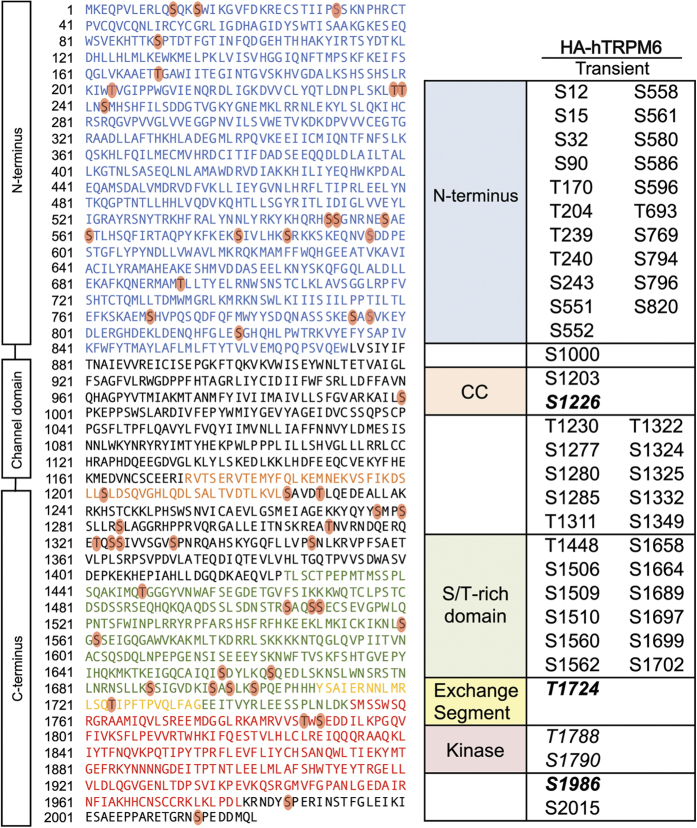
Identification of TRPM6 phosphorylation sites. The diagram on the left depicts the amino acid sequence of human TRPM6 (UniProtKB: Q9BX84). The cytosolic N-terminus, the coiled-coil domain, the S/T-rich domain, the exchange segment, and the alpha-kinase domain are colored in blue, orange, green, yellow and red respectively. Red circles highlight phosphorylated residues identified on hTRPM6. The chart on the right summarizes all of the phosphorylation sites identified in this analysis. Residues in bold italics represent TRPM6 residues whose conserved counterparts on TRPM7 were also phosphorylated.

It has been reported that TRPM7 can be phosphorylated by TRPM6 and that the phosphorylation events may be important for intracellular trafficking of TRPM7^28^,^29^. To identify potential transphosphorylation sites on TRPM7 by TRPM6, we employed a kinase-inactive mouse TRPM7 mutant tagged with streptavidin binding protein (SBP-TRPM7-K1646R) expressed alone in HEK-293T cells or co-expressed with wild-type HA-tagged human TRPM6 (HA-TRPM6). *In vitro* kinase assays verified the respective kinase activity of SBP-TRPM7-K1646R and HA-TRPM6 proteins (Fig. 1e,f), and a co-immunoprecipitation assay confirmed that TRPM6 and TRPM7-K1646R assemble into a complex when co-expressed in HEK-293T cells (Fig. 1g). The mass spectrometric analysis uncovered a large number of transphosphorylation sites on the kinase-inactive TRPM7 that were introduced by co-expressed TRPM6 (Fig. 3). Seven transphosphorylation residues found on the kinase-inactive TRPM7 were previously identified as TRPM7 autophosphorylation sites (TRPM7 S1224, S1255, S1299, S1403, S1565, S1567, and S1693). In this analysis, S1255 and S1567 were the two most prominently transphosphorylated sites (Supplementary Table 4). TRPM7 had been reported to not phosphorylate TRPM6^28^,^29^. Our experiments indicate that this transphosphorylation reaction does occur but is not robust as TRPM6 autophosphorylation (Supplementary Fig. 1). We thus proceeded with an analysis of TRPM6 autophosphorylation sites. Our MS analysis indeed revealed extensive phosphorylation of TRPM6 when the HA-tagged human TRPM6 (HA-TRPM6) was individually expressed in HEK-293T cells (Fig. 4). Interestingly, we found three TRPM6 phosphorylation sites analogous to residues on TRPM7 that are also phosphorylated (TRPM6 S1226, T1724, S1986 versus TRPM7 T1250, S1567 and T1828) (Fig. 4). Surprisingly, unlike TRPM7 the most prominently phosphorylated residue on TRPM6 was on its NH_2_-terminus (S552) (Supplementary Table 5).

### TRPM7 kinase activity is affected by autophosphorylation

As some the autophosphorylation sites we identified occurred within TRPM7’s kinase domain, this prompted us to investigate more closely the impact of phosphorylation on TRPM7 kinase’s catalytic activity. TRPM7 purified from mammalian cells revealed only a limited number of phosphorylation sites on the catalytic core domain. To ensure that we identified all potential autophosphorylation sites, we purified a Sumo-tagged mouse TRPM7 kinase (a.a. 1384–1863) (Sumo-TRPM7-Kinase) (Fig. 1a) from bacteria and stimulated the kinase with ATP *in vitro* before MS analysis. A catalytically inactive mutant of TRPM7 kinase was also analyzed as a negative control (Fig. 1h)*. In vitro,* ATP stimulation enriched the number of autophosphorylation sites identified (Fig. 2 and Supplementary Table 6; representative MS/MS shown in Supplementary Fig. 2), and in particular, revealed five additional phosphorylation sites within the kinase’s catalytic core domain.

**Figure 5:**
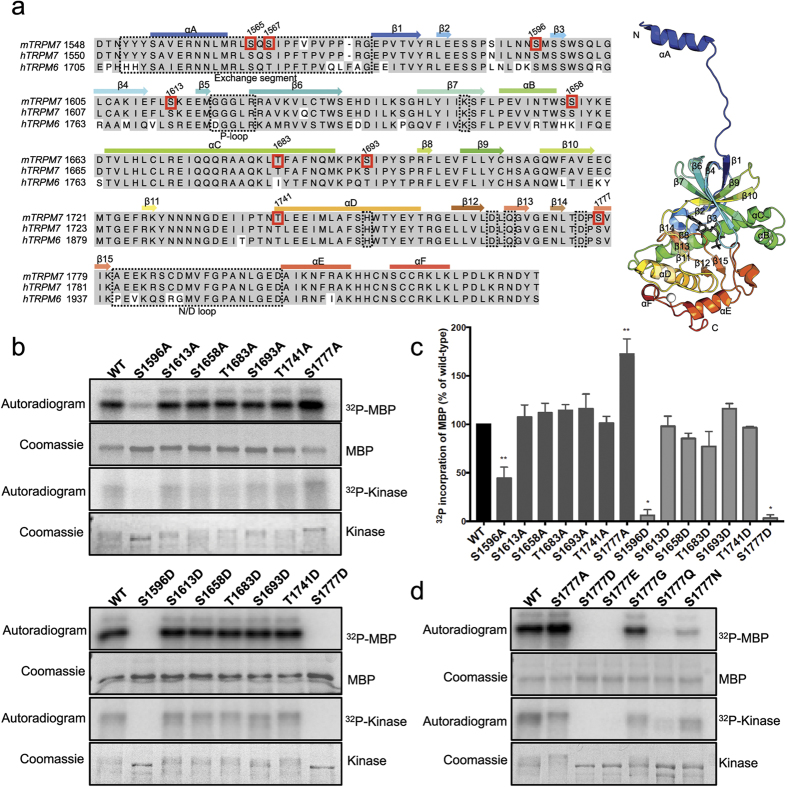
Mutagenesis screen of TRPM7 kinase domain phosphorylation sites. (**a**) Sequence alignment of mouse TRPM7, human TRPM7, and human TRPM6 (UniProtKB: Q923J1, Q96QT4, and Q9BX84). The position of α-helices (boxes) and β-strands (arrows) are shown above the alignment. Functionally important motifs and resides on the mouse TRPM7 sequence are shown in dashed boxes. Seven identified autophosphorylation residues on the mouse TRPM7 sequence are shown in red boxes. On the right, a ribbon diagram depicts the structure of mouse TRPM7 kinase domain (PDB code 1IA9). The AMPPNP is rendered as gray sticks and the zinc atom as a white sphere. The N- and C-termini are indicated as “N” and “C”. (**b**) Sumo-TRPM7-Kinase WT and mutants were purified from *E. Coli* as described in the Materials and Methods. Catalytic actives of the WT and mutant kinases were assessed in *in vitro* kinase assays using MBP as a substrate. The kinase assays were performed at 30 °C for 2 min. (**c**) Quantification of ^32^P incorporation into MBP is shown as a histogram. *P < 0.05, **P < 0.01. Results are means ± S.E.M (n = 2–9). (**d**) The catalytic activities of Sumo-TRPM7-Kinase WT and various S1777 mutants were assessed by an *in vitro* kinase assay as described in the Materials and Methods. The kinase assays were performed at 30 °C for 2 min.

**Figure 6:**
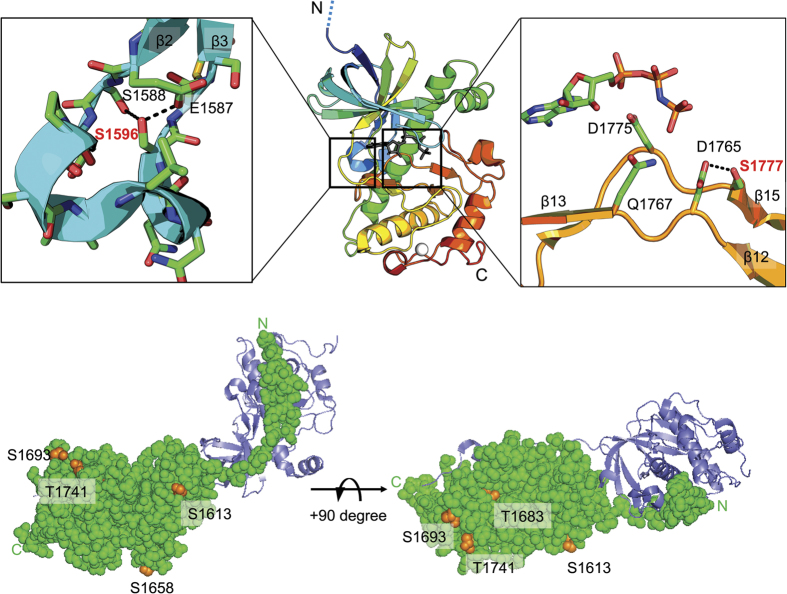
Structural analysis of autophosphorylated residues on TRPM7 kinase domain. (**a**) Ribbon diagram in the center shows the structure the core catalytic domain (a.a. 1575–1828) of mouse TRPM7 kinase (PDB code 1IA9). The AMPPNP is rendered as gray sticks and the zinc atom as a white sphere. The N- and C-termini are indicated as “N” and “C”. The expanded view on the left highlights the location of residue S1596 on an omega-loop between the β2 and β3 strands. Side chains of residues are shown as sticks. Interactions between residues S1596, S1588 and E1587 are indicated with dashed lines. The expanded view on the right shows the position of residue S1777 at the active site along with catalytic residues D1765, Q1767, and D1775. The nucleotide and side-chains of key residues are shown as sticks. The interaction between S1777 and D1765 is shown with a dashed line. (**b**) Diagram depicts dimerized TRPM7 kinase. Space-filling model shows the location of residue S1613, S1658, T1683, S1693, and T1741 (orange) on the surface of kinase monomer A (green). The N- and C-termini of the monomer are indicated as “N” and “C”. A dimerizing monomer B is shown as the ribbon in purple.

To assess the importance of the phosphorylated residues on TRPM7 kinase domain to its catalytic activity we conducted a mutagenesis screen. The five autophosphorylated sites identified on the Sumo-TRPM7-Kinase (S1658, T1683, S1693, T1741, and S1777), S1613, which was discovered on the constitutively expressed FLAG-TRPM7, and S1596, a phosphosite identified in a previous study^24^, were mutated to either non-phosphorylatable alanine or the phosphomimetic residue aspartate (Fig. 5a). The catalytic activity of WT and mutants were then tested using *in vitro* kinase assays. Our screen identified two autophosphorylation sites S1596 and S1777 on the kinase catalytic core domain that influenced catalytic activity (Fig. 5b,c). We found that either substitution at S1596 to alanine or aspartate compromised catalytic activity. Close examination of kinase crystal structure reveals that S1596 is located on an omega-loop between the β2 and β3 strand, with its side-chain hydroxyl group forming hydrogen bonds with S1588 and E1587 (Fig. 6a). Omega-loops are hydrogen bond-rich non-regular secondary structures that are often found on the surface of globular proteins and involved in protein folding and stability^33^. Substitution of residues at this site likely affected the structural stability of kinase, compromising catalytic activity as a result. By contrast, we found the non-phosphorylatable alanine substitution at S1777 significantly increased TRPM7’s kinase activity while phosphomimetic mutations to either glutamate or aspartate abolished kinase activity (Fig. 5b,c). S1777 is located near the catalytic centre of kinase and forms a hydrogen bond with D1765 (Fig. 6a), the invariant key catalytic aspartate residue responsible for properly orientating the hydroxyl group of the substrate towards the γ-phosphate of ATP for catalysis^15^,^16^. Taking into account the proximity of S1777 to the active site of the TRPM7 kinase, we hypothesized that the negative charge introduced by S1777 phosphorylation could cause a perturbation of the overall electrostatic interactions at the catalytic centre and disrupt catalysis. Another possibility we considered is that addition of the large phosphate group on S1777 could be impeding the binding of ATP to the catalytic core, whereas the S1777A substitution would prevent such a modification and also better accommodate ATP for hydrolysis and phosphate transfer. We mutated S1777 to either asparagine (S1777N) or glutamine (S1777Q), two uncharged polar residues that structurally resemble their charged aspartate and glutamate counterparts, and found that these substitutions significantly compromised kinase activity (Fig. 5d). By contrast, mutating S1777 to glycine (S1777G), which possesses the smallest side chain and is also non-phosphorylatable produced a kinase that retained kinase activity (Fig. 5d). This result indicates that functional catalysis by TRPM7 kinase requires an amino acid at position 1777 with a relatively small side chain. Thus, the addition of a phosphate group at S1777 by phosphorylation likely disrupts catalysis.

**Figure 7:**
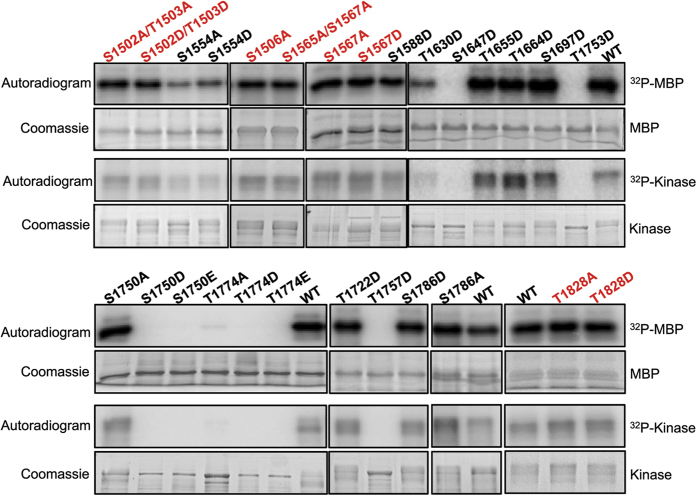
Mutagenesis screen of serine and threonine residues on TRPM7 with regulatory potential. The indicated Sumo-TRPM7-Kinase mutants were purified from *E. Coli*, and their kinase activities were assayed in *in vitro* kinase assays using MBP as a substrate. The kinase assays were performed at 30 °C for 2 min. Proteins were resolved by SDS-PAGE and subjected to Coomassie blue staining. Phosphorylation was detected by autoradiography. Residues highlighted in red are the ones found to be phosphorylated by mass spectrometry.

Substitution of alanine or aspartate at the five other serine and threonine residues (S1613, S1658, T1683, S1693, and T1741) did not cause a significant change in the catalytic activity. Close examination of the kinase structure found that these five serine and threonine residues are exposed at the protein’s surface (Fig. 6b), suggesting that substitutions at this site did not cause structural disruption and that these residues likely do not function as regulatory sites for the kinase. To complete our investigation of factors that may modulate TRPM7’s catalytic activity within the core domain, we explored whether serine or threonine residues not subject to autophosphorylation, but rather located close to functionally important residues of the kinase, could regulate the kinase activity when they become phosphorylated. Residue S1750 is located adjacent to the invariant zinc-binding coordinating residue H1751 on the α-helix D of the TRPM7 kinase, along with two other highly conserved residues T1753 and T1757 (Fig. 5a). Substitution of S1750, T1753 and T1757 to the phosphomimetic aspartate and/or glutamate abolished catalytic activity (Fig. 7). Analysis of the TRPM7 kinase structure indicated that phosphomimetic substitution of residues on this α-helix might disrupt the overall protein stability. Meanwhile, substitutions at T1774, which is adjacent to the invariant catalytic residue D1775, to alanine, aspartate, or glutamate, also produced severe disruption to the catalytic activity (Fig. 7). Previous studies found that a threonine to serine substitution at this site also inactivated the kinase, suggesting the importance of the threonine side chain in mediating the catalysis^16^,^34^. We also found that aspartate substitution of S1647, which is adjacent to the invariant catalytic K1646, caused kinase inactivation (Fig. 7). The fact that those serine and threonine residues are never found to be phosphorylated suggests that they are not likely to be regulatory sites.

As many conventional kinases and alpha-kinases are subject to regulation by phosphorylation by other kinases, we explored whether serine or threonine residues contained within phosphorylation motifs of other kinases have the potential to influence TRPM7 kinase activity. For example, we found that S1588 resides within the phosphorylation motif of NIMA-related kinase-6, S1554 and T1630 are within a casein kinase 2 phosphorylation motif, and T1664 is within a motif for proline-directed kinases^35^. We generated phosphomimetic substitutions at those sites (S1554A/D, S1558D, T1630D, and T1664D), and also at a few other non-phosphorylated serine/threonine residues within the kinase domain (T1655D, S1697D, T1722D and S1786A/D), but found no change in the kinase’s catalytic activity. As members of the alpha-kinases family have regulatory phosphorylation sites outside the catalytic core^26^,^27^, we looked at phosphorylated residues S1502, T1503, S1506, and T1828 that are outside of TRPM7 kinase domain. However, substitution of alanine or aspartate at these sites also had no effect on the kinase’s catalytic activity (Fig. 7). One known interacting protein of TRPM7 kinase is phospholipase C beta-1 (PLC-β1)^36^. The C2 domain of PLC-β1 binds to the last 146 amino acids of TRPM7’s kinase domain, but itself is not a substrate of TRPM7’s kinase^36^,^37^. Rather, activation of PLC leads to inactivation of the channel^37^. To answer the question of whether binding of PLC-β1 to the channel’s kinase domain has any impact the kinase’s catalytic activity, we affinity purified PLC-β1 and employed it in an *in vitro* kinase assay. We found that PLC-β1 does not impact the kinase’s catalytic activity towards the MBP substrate (Supplementary Fig. 3).

### Phosphorylation of the TRPM7 kinase exchange segment affects catalytic activity

**Figure 8:**
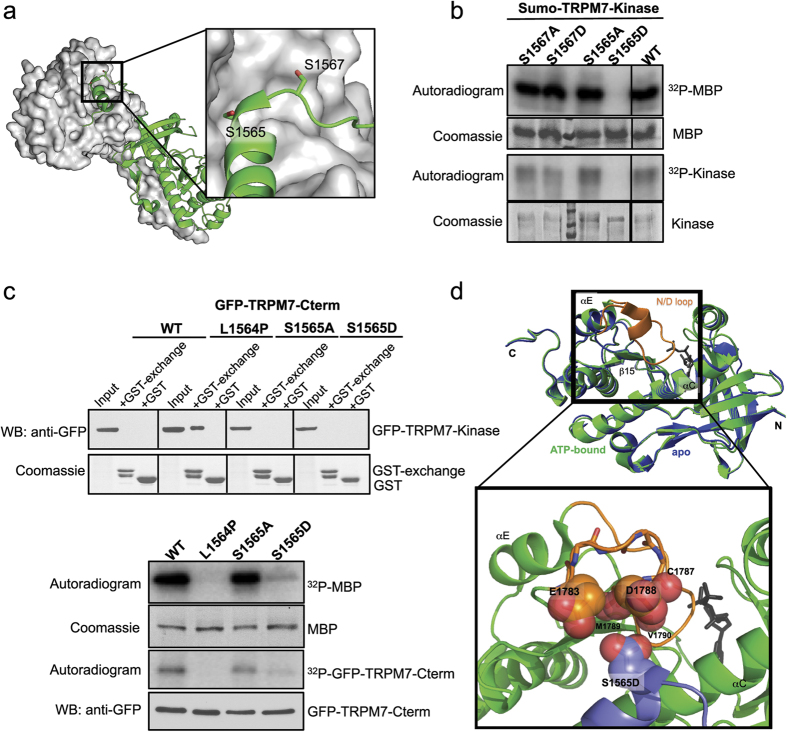
S1565 in the TRPM7 kinase dimerization exchange segment regulates catalytic activity. (**a**) The structure of a dimerized TRPM7 kinase depicts the N-terminal exchange segment of one kinase monomer (ribbon diagram in green) interacting with another one (space-filled surface in gray). The expanded view on the right highlights the location of S1565 and S1567 on the exchange domain of one kinase monomer (side-chains shown as sticks). (**b**) An *in vitro* kinase assay assesses the catalytic activities of Sumo-TRPM7-Kinase WT and mutants carrying S1565A, S1565D, S1567A, and S1567D substitutions. The kinase assay was performed at 30 °C for 2 min. (**c**) GFP-TRPM7-Cterm containing WT kinase or mutants harboring the L1564P, S1565A, and S1565D substitutions were expressed in HEK-293T cells. Proteins in the lysates were affinity purified by GST-tagged mTRPM7 exchange segment (a.a. 1548–1576) (GST-exchange) bound to glutathione agarose. The affinity purified samples were resolved by SDS-PAGE and Coomassie blue staining. GFP-TRPM7-Cterm proteins were analyzed by immunoblotting with an anti-GFP antibody (top). An *in vitro* kinase assay was carried out to validate the catalytic activity of GFP-TRPM7-Cterm WT and mutant kinases. The kinase reaction was performed at 30 °C for 20 min. The presence of purified kinases was validated by western blotting using an anti-GFP antibody (bottom). (**d**) Overlaying image of the TRPM7 kinase in the ATP-bound state (PDB: 1IA9, green) and the apo-state (PDB: 1IAJ, blue). Orange color highlights the flexible N/D loop (a.a. 1783–1798) that is missing in the apo-state. The AMPPNP is shown as gray sticks. The zoomed panel at the bottom shows the flexible N/D loop (orange) of one kinase monomer superimposed with the exchange segment of another monomer containing the S1565D mutation (purple). Side chain carboxylates of D1565, E1783, and D1788, and backbone carbonyls of C1787, M1789, and V1790 are shown as red spheres.

A distinguishing feature of TRPM7 is that the kinase domain forms a dimer in the crystal structure as a consequence of the exchange between monomers of an NH_2_-terminal segment that is mainly helical (a.a. 1551–1577) (Figs 5a and 8a)^15^. This exchange segment is divided into two functional motifs: an “activation motif” (a.a. 1553–1562) that is essential for kinase activity and makes direct contacts with residues in the kinase domain of the other TRPM7 monomer; and also a “dimerization motif” (a.a. 1563–1570) that is critical for the functional dimer assembly^17^. Within the “dimerization motif” of TRPM7, two serine resides (S1565 and S1567) have been identified as autophosphorylation sites by our screen and other studies^20^,^25^. To determine whether phosphorylation of S1565 and S1567 could potentially affect kinase activity, we mutated these serine residues to alanine and aspartate. We observed that mutation of S1565 to the phosphomimetic amino acid aspartate (S1565D) abolished catalytic activity, whereas when S1565 was changed to alanine (S1565A) there was no effect (Fig. 8b). Mutation of residue S1567 to either alanine or aspartate (S1567A and S1567D), however, had no effect on the kinase’s catalytic activity (Fig. 8b). Previous studies have shown that mutation of the L1564 adjacent to the S1565 on the “dimerization motif” abolished kinase catalytic activity by disrupting dimer formation^17^. We, therefore, speculated that the phosphomimetic substitution S1565D might similarly disrupt catalytic activity by interfering with TRPM7 kinase dimerization. We purified GFP-tagged C-terminal fragment of wild-type mTRPM7 kinase (GFP-TRPM7-Cterm-WT) and kinases harbouring L1564P, S1565A and S1565D mutations (GFP-TRPM7-Cterm-L1564P, S1565A, and S1566D). *In vitro* kinase assay using MBP as a substrate indeed confirmed the catalytic defects for both L1564P and S1565D mutants (Fig. 8c). To test for disruption of dimer formation, we performed an *in vitro* GST pulldown assay using GST-tagged TRPM7 exchange domain containing residue 1548–1576 (GST-exchange) and investigated its ability to affinity purify GFP-TRPM7-Cterm WT and mutants from cell lysate (Fig. 8c). If dimer formation of the TRPM7 kinase were disrupted, GST-exchange would be able to affinity purify the kinase. We found GST-exchange was not able to affinity purify the WT TRPM7-Cterm, presumably because the dimer interaction between the monomers could not be overcome. By contrast, GST-exchange successfully affinity purified GFP-TRPM7-Cterm-L1564P, indicating that the L1565P mutation disrupted the stability of the dimer. Surprisingly, the introduction of S1565A or S1565D mutations into TRPM7-Cterm did not disrupt dimerization, as assessed by the inability of GST-exchange to affinity purify GFP-TRPM7-Cterm-S1565A and S1565D proteins. This data indicates the S1565D mutation in TRPM7’s exchange segment disables kinase catalytic activity without significantly interfering with dimerization, suggesting that phosphorylated S1565 may behave similarly.

One explanation for the catalytic defect observed in S1565D mutant comes from the observation that the side chain of S1565 is orientated towards a glycine-rich loop (a.a. 1783–1798) between the β15 sheet and the αE strand of the other monomer. This loop of alpha-kinases is referred to as the “N/D loop” due to the presence of invariant asparagine or aspartate residue^38^. The N/D loop of TRPM7 is highly flexible as it is absent in the crystal structure of the kinase without ATP (Fig. 8d)^15^. S1565 in the exchange domain of one TRPM7 kinase monomer faces a wall of negative charges from the side chain carboxylates of E1783 and D1788, and the backbone carbonyls of residues C1787, M1789, and V1790 in the flexible N/D loop of the other TRPM7 kinase monomer (Fig. 8d). Molecular modeling indicates that the ATP-binding site at the kinase catalytic core does not have direct contacts with S1565 that would be perturbed upon S1565 mutation to aspartate. Rather, substitution of aspartate at S1565 would introduce a negative charge, which would cause electrostatic repulsion of the N/D loop away from its original location. Studies of this N/D loop in other alpha-kinases have suggested its involvement in the substrate binding^26^. Moreover, by comparing the crystal structure of the MHCK A to the TRPM7 kinase structure, Ye and colleagues speculated that “the N/D loop not only dictates the size and shape of the active-site pocket but may also act as a regulatory switch to control access to it”^38^. We, therefore, speculate that phosphorylation of S1565 changes the structure of the N/D loop, disrupting the ability of the kinase to bind to its substrate.

## Discussion

With the aim of understanding how phosphorylation regulates the two unique channel-kinases TRPM6 and TRPM7, our study deployed LC-MS/MS to survey phosphorylation sites present on these two proteins using various constructs, examining phosphorylation *in vivo* and *in vitro.* One of our concerns was that which sites on TRPM7 are phosphorylated *in vivo* might vary depending on whether the full-length protein is present on the cell surface or located in intracellular compartments such as the ER, which is where the channel is predominantly found when transiently overexpressed in HEK-293T cells. Also, our previous studies found that overexpression of TRPM7 causes cell stress, which could also potentially influence the phosphorylation state of TRPM7^39^. Indeed, the phosphorylation pattern detected for full-length mTRPM7 protein transiently overexpressed differed from the one obtained for constitutively and more lowly expressed TRPM7. Our MS analysis identified S1567 as a prominent autophosphorylation site on the overexpressed full-length TRPM7 and the COOH-terminal fragment, which is in agreement with the previous findings^24^,^25^,^34^. However, S1360 was the most frequently phosphorylated residue found on the constitutively expressed full-length protein. This difference suggests that phosphorylation of different sites may have a regulatory impact on the channel-kinase during the dynamic process of protein biosynthesis and trafficking. We also found that the phosphorylation pattern of TRPM7 was affected by whether TRPM7’s kinase is attached to the ion channel domain. Proteolytic cleavage of TRPM7 by caspases releases a catalytically active form of the COOH-terminal kinase domain beginning at D1510, and several COOH-terminal fragments of TRPM7 have been identified in different cell types and tissues and functionally linked to the regulation of chromatin remodelling^23^,^32^. For the liberated COOH-terminal fragments of the kinase, its phosphorylation may impact kinase trafficking or the function of the kinase within the nucleus.

Previous studies have reported that TRPM6 can cross-phosphorylate TRPM7 but not vice versa^29^,^30^. Our experiments indicate, however, that modest transphosphorylation of TRPM6 by TRPM7 can occur, but the physiological significance of this event remains unknown. It has also been reported that TRPM6 requires co-expression of TRPM7 to be recruited to the plasma membrane^4^,^28^,^40^,^41^. It is possible that transphosphorylation of TRPM7 by TRPM6 produces trafficking signals to direct the assembled TRPM6/TRPM7 complex to the cell surface. We speculate that this trafficking signal may be initiated by phosphorylation of site(s) that are exclusive to TRPM7 and that these site(s) have no phosphorylatable analogues in TRPM6. This is one possible explanation for why autophosphorylation of TRPM6 by itself fails to initiate the process of cell surface trafficking. Such a trafficking-associated phosphorylation signal on TRPM7 may also explain the previous observation that TRPM7 intracellular localization is altered upon co-expression of TRPM6^29^. Taken together, these data provide insights into possible mechanisms by which the kinase activities of TRPM6 and TRPM7 may regulate the intracellular tracking and localization of the proteins.

To further understand how phosphorylation events could be controlling the catalytic activity of TRPM7, we closely examined multiple serine and threonine residues near or in the kinase domain and uncovered a number of autophosphorylated sites whose mutation affected catalytic activity. S1777 is highly conserved in TRPM7 and TRPM6 (but not in other alpha-kinases) and located at the active site close to key catalytic residues. The addition of a phosphate group to S1777 is likely to break the existing hydrogen bond between S1777 and the key catalytic residue D1765 and introduce a negative charge in the vicinity of ATP. It is possible that S1777 phosphorylation functions as a stop signal for TRPM7 catalysis, though it is not clear whether this “off” signal could be easily reversed given its buried location. By contrast, S1565 is located outside of the TRPM7 catalytic core on the kinase’s exchange domain. Though phosphorylation of residues on the exchange domain does not affect TRPM7 kinase dimer formation, close examination of the kinase structure suggests a possible interaction between S1565 of one kinase monomer and the flexible N/D loop of the other monomer. The N/D loop of alpha-kinases is considered to be equivalent to the activation loop of conventional serine/threonine kinases, which is located at the bottom of large C-lobe facing toward the catalytic cleft^15^,^38^. For conventional kinases, phosphorylation of the activation loop stabilizes the protein in an active conformation more permissive to substrate binding, serving as an important regulatory mechanism to control the kinase activity^42^. The N/D loops of alpha-kinases, in contrast, do not appear to be phosphorylated. However, they are still critical for the kinase activity as they have been speculated to participate in substrate binding and to control the access of the substrate to the active sites of MHCKs and eEF2K^26^,^38^,^43^. Our experiments and molecular modeling indicate that S1565 on the exchange domain helix is orientated toward a negatively charged surface of the N/D loop in the TRPM7 kinase dimer. Phosphorylation of S1565 would cause electrostatic repulsion of the N/D loop away from S1565, which could adversely affect substrate accessibility to the active site of the kinase. As TRPM7 S1565 was identified as both autophosphorylated and transphosphorylated site in our MS analysis, we propose that S1565 phosphorylation within the exchange domain functions as a regulatory switch to control TRPM7 kinase activity through elicited movements of the N/D loop.

Our current study has attempted to understand how regulation of TRPM6 and TRPM7 kinase activity differs from that of the other alpha-kinases such as MHCKs and eEF2K. A comparison between the crystal structures of MHCK A and TRPM7 shows a high degree of similarity in the core catalytic domain, but while TRPM6 and TRPM7 kinases form dimers, the kinase domain of MHCK A is a functional monomer and does not possess an exchange segment homologous to that of TRPM6 and TRPM7^38^. Also, previous investigations of MHCKs and eEF2K identified a phospho-specific allosteric binding site (Pi-pocket) proximal to the active site^26^,^38^. Autophosphorylation of a conserved threonine residue on the COOH-tail of the kinases (MHCK A T825 and eEF2K T348) creates a phosphate ligand for this Pi-pocket, eliciting a conformational change in the catalytic center that stimulates MHCK A and eEF2K catalytic activity^26^,^27^,^38^,^44^. TRPM6 and TRPM7 do not appear to have a similar Pi-pocket^15^,^38^,^45^. Even though conserved phospho-threonine/serine residues are present on the COOH-terminal tail of both kinases (TRPM7 T1828 and TRPM6 S1986), our study has shown that substitution of alanine or aspartate for TRPM7 T1828 does not affect catalytic activity. This result indicates that TRPM7 and TRPM6 kinases operate under a different mode of regulation from MHCKs and eEF2Ks.

In this study, we performed a compressive analysis of phosphorylation sites present on TRPM6 and TRPM7 as a first step towards understanding the regulatory mechanisms of these channel-kinases. Indeed, our discovery that phosphorylation of TRPM7 S1565 inactivates kinase activity without affecting dimerization provides a unique mechanism by which *in vivo* TRPM7 kinase activity can be controlled. Future studies will be directed at determining whether phosphorylation of S1565, as well as S1777, plays a functional role in regulating TRPM7 kinase activity *in vivo*. Now that phosphorylation sites on both TRPM6 and TRPM7 have been identified, we can also begin the work of teasing apart how phosphorylation of these unique channel-kinases impacts their functions.

## Methods

### Constructs

The GFP-TRPM7-Cterm (a.a. 1120–1863) containing a streptavidin binding protein (SBP) tag was generated in an earlier study^11^. To generate the Sumo-TRPM7-Kinase, mouse TRPM7 cDNA (a.a. 1384–1863) was amplified by PCR and subcloned into the *BamHI/EcoRI* sites of a pTrcHis2 A vector (Thermo Fisher Scientific, MA) modified to contain a NH_2_-terminal His6-Sumo-SBP tag between *NcoI/BamH1*. Site-directed point mutations were introduced into TRPM7 using the QuickChange mutagenesis kit (Stratagene, CA). To generate the GST-fused exchange peptide of TRPM7, residues 1548–1576 of mouse TRPM7 were amplified by PCR and fused in-frame to GST by cloning into the *BamHI* and *EcoRI* cloning sites of the pGEX-6P3 vector (Addgene, MA). The kinase-inactive TRPM6-K1804R mutant was generated by site-directed mutagenesis (QuickChange; Stratagene, CA) using the HA-tagged human TRPM6 as a template.

### Cell lines

The 293 T cell line (CRL-3216) was purchased from American Type Culture Collection (Manassas, VA). The HEK-293-TRPM7 cells expressing recombinant FLAG-tagged mouse TRPM7 was described in an earlier publication^31^. HEK-293T and HEK-293-TRPM7 cells were maintained in a Dulbecco’s Modified Eagle Medium, high glucose media with 10% fetal bovine serum in a humidified 37 °C, 5% CO_2_ incubator.

### Expression and purification of TRPM7 kinases

To identify autophosphorylation sites of TRPM7, HA-TRPM7 WT and K1646R and GFP-TRPM7-Cterm WT and K1646R were transiently expressed in HEK-293T cells. Cells were lysed with lysis buffer containing 50 mM Tris (pH 7.4), 150 mM NaCl, 1% IGEPAL CA-630 (Sigma-Aldrich, MO), protease inhibitor cocktail (Roche Life Sciences, IN), and phosphatase inhibitor cocktail (EMD Millipore, Germany). HA-TRPM7 proteins were immunoprecipitated by HA-agarose (Roche Life Sciences, IN) and eluted with 1 mg/ml HA-peptide (Roche Life Sciences, IN). The GFP-TRPM7-Cterm proteins containing an SBP tag was immunoprecipitated by streptavidin resin (Stratagene, CA) and eluted with 10 mM biotin (Sigma, MO). FLAG-tagged TRPM7 was obtained from the HEK-293-TRPM7 cell line expressing recombinant mouse FLAG-TRPM7 at a basal level without tetracycline induction and immunoprecipitated by M2 FLAG-agarose (Sigma-Aldrich, MO)^31^. To identify cross-phosphorylation sites on TRPM7, a kinase-inactive mouse SBP-TRPM7-K1646R was co-expressed with HA-tagged human TRPM6 in HEK-293T cells, immunopurified by HA-agarose, and eluted with 1 mg/ml HA-peptide. As a negative control, SBP-TRPM7-K1646R was expressed alone in HEK-293T cells, immunopurified by streptavidin agarose, and eluted with 10 mM biotin. To identify TRPM6 phosphorylation sites, HA-hTRPM6 proteins transiently expressed in HEK-293T cells were immunoprecipitated by HA agarose and eluted with 1 mg/ml HA-peptide. The Sumo-TRPM7-Kinase was expressed in transformed *E. Coli* BL21-DE3 cells (Stratagene, CA). Bacteria were grown at 37 °C to OD600 of 0.6–0.8, cooled to 16 °C and induced at 16 °C for 16–18 h with 1 mM isopropyl-beta-D-thiogalactopyranoside (Gold Biotechnology, MO). Cells were lysed by sonication in ice-cold phosphate buffered saline (PBS) containing a final concentration 1 mM protease inhibitor PMSF and 1% Triton-X10. Sumo-TRPM7-Kinase was pulled down from the cell lysate supernatant by streptavidin agarose (Stratagene, CA) and eluted with 10 mM Biotin (Sigma, MO). Before MS analysis, purified Sumo-TRPM7-Kinase WT and KR was stimulated with 0.5 mM ATP in kinase buffer (50 mM MOPS (pH 7.2), 100 mM NaCl, 2.5 mM MnCl_2_) at 30 °C for 20 min.

### Phosphorylation site identification by LC-MS/MS

For phosphorylation site identification, purified TRPM6 and TRPM7 were resolved in a Bis-Tris polyacrylamide gel, and each gel band was subjected to in-gel reduction, alkylation, tryptic digestion and peptide extraction with a standard protocol. Peptides were solubilized in 0.1% trifluoroacetic acid, and analyzed by Nano LC-MS/MS with Dionex Ultimate 3000 RLSC Nano System interfaced with a Velos-LTQ-Orbitrap (ThermoFisher, CA) or a QExactive HF (Thermofisher, CA) based on instrument availability. Samples were loaded onto a self-packed 100 μm × 2 cm trap (Magic C18AQ, 5 μm 200 Å; Michrom Bioresources, Inc., PA) and washed with Buffer A (0.2% formic acid) for 5 min with a flow rate of 10 μl/min. The trap was brought in-line with the analytical column (Magic C18AQ, 3 μm 200 Å, 75 μm × 50 cm; Michrom Bioresources, Inc., PA) and peptides fractionated at 300 nL/min using a segmented linear gradient 4–15% Buffer B (0.2% formic acid in acetonitrile) in 35 min, 15–25% Buffer B in 65 min, 25–50% Buffer B in 55 min. Mass spectrometry data was acquired using a data-dependent acquisition procedure with a cyclic series of a full scan acquired with a resolution of 60,000 (Velos-LTQ-Orbitrap) or 120,000 (QExactive HF) followed by MS/MS of the 20 most intense ions and a dynamic exclusion duration of 30 sec for both instruments. Proteome Discoverer was used for database search and analysis. Data were searched against most updated SwissProt database using MASCOT (v 2.3). Precursor ion mass error tolerance was set to ±10 ppm (Velos-LTQ-Orbitrap) or +/− 7 ppm (QExactive HF) and fragment mass error tolerance to ±0.4 Da (Velos-LTQ-Orbitrap) and +/−20 ppm (QExactive HF). Cysteine carbamidomethylation was set as a complete modification, acetylation on N-terminus of protein, oxidation on methionine, phosphorylation on serine, threonine and tyrosine were set as variable modifications. Site localization was analyzed using PTMRS. Only spectra of high confidence (FDR were set at 0.01 for PSM) were reported. Interested sites were further validated manually.

### Kinase assay

Purified TRPM7 proteins were dialyzed into kinase buffer (50 mM MOPS (pH 7.2), 100 mM NaCl, 2.5 mM MnCl_2_, 0.5 mM ATP). The kinase reactions were performed at 30 °C for various times in the presence of 4 μCi of [γ-^32^P]ATP with 5 μg of myelin basic protein as a substrate. Reactions were stopped by the addition of SDS sample buffer, and the reaction mix was resolved by SDS-PAGE. Proteins were detected by Coomassie blue staining and gels were dried completely using a gel dryer (BioRad Laboratories, CA). Incorporation of [γ-^32^P]ATP into substrates was analyzed by autoradiography using Cyclone Plus Phosphor Imager (PerkinElmer, CT).

### GST pull-down assays

Recombinant GST-exchange peptide and GST were expressed in *E. Coli* BL21-DE3 cells (Agilent Technologies, CA) and purified from cell lysates by incubating with glutathione agarose (Sigma, MO) at 4 °C for overnight. The resin was washed thoroughly with PBS with 1% Tween-20. For GST pull-down, HEK-293T cells lysate containing transiently expressed GFP-TRPM7-Cterm was incubated with GST-exchange peptide or GST proteins bound on glutathione agarose for overnight at 4 °C.

### Immunoblotting

For detection of proteins, the proteins resolved by SDS-PAGE and western blotting using standard protocols. A rat monoclonal antibody anti-HA antibody (3F10; Roche Life Sciences, IN) was used to detect HA-tagged TRPM7 and TRPM6. Anti-FLAG M2 antibody (Sigma, MO) was used to detect FLAG-TRPM7. A mouse monoclonal antibody anti-SBP (sc-101595; Santa Cruz Biotechnology, Inc., TX) and a rabbit polyclonal anti-TRPM7-C47 were used to detect SBP-TRPM7^7^. An anti-GFP antibody (sc-9996; Santa Cruz Biotechnology, Inc., TX) was used to detect GFP-TRPM7-Cterm. An anti-pSer antibody (#2981; Cell Signalling Technology, Inc., MA) was used to detected phosphorylated TRPM6 protein.

## Additional Information

**How to cite this article**: Cai, N. *et al*. Mass Spectrometric Analysis of TRPM6 and TRPM7 Phosphorylation Reveals Regulatory Mechanisms of the Channel-Kinases. *Sci. Rep.* **7**, 42739; doi: 10.1038/srep42739 (2017).

**Publisher's note:** Springer Nature remains neutral with regard to jurisdictional claims in published maps and institutional affiliations.

## Supplementary Material

Supplementary Information

## References

[b1] Chubanov V. . Hypomagnesemia with secondary hypocalcemia due to a missense mutation in the putative pore-forming region of TRPM6. J Biol Chem. 282, 7656–7667 (2007). 17197439 10.1074/jbc.M611117200

[b2] Lainez S. . New TRPM6 missense mutations linked to hypomagnesemia with secondary hypocalcemia. Eur J Hum Genet. 22, 497–504 (2014). 23942199 10.1038/ejhg.2013.178 PMC3953905

[b3] Schlingmann K. P. . Hypomagnesemia with secondary hypocalcemia is caused by mutations in TRPM6, a new member of the TRPM gene family. Nat Genet. 31, 166–170 (2002). 12032568 10.1038/ng889

[b4] Chubanov V. . Disruption of TRPM6/TRPM7 complex formation by a mutation in the TRPM6 gene causes hypomagnesemia with secondary hypocalcemia. PNAS. 101, 2894–2899 (2004). 14976260 10.1073/pnas.0305252101 PMC365716

[b5] Ryazanova L. V. . TRPM7 is essential for Mg(2+) homeostasis in mammals. Nat Commun. 1, 109 (2010). 21045827 10.1038/ncomms1108 PMC3060619

[b6] Schmitz C. . Regulation of vertebrate cellular Mg2+ homeostasis by TRPM7. Cell. 114, 191–200 (2003). 12887921 10.1016/s0092-8674(03)00556-7

[b7] Su L. T. . TRPM7 regulates cell adhesion by controlling the calcium-dependent protease calpain. J Biol Chem. 281, 11260–11270 (2006). 16436382 10.1074/jbc.M512885200 PMC3225339

[b8] Su L. T. . TRPM7 regulates polarized cell movements. Biochem J. 434, 513–521 (2011). 21208190 10.1042/BJ20101678 PMC3507444

[b9] Decker A. R. . Abnormal differentiation of dopaminergic neurons in zebrafish trpm7 mutant larvae impairs development of the motor pattern. Dev Biol. 386, 428–439 (2014). 24291744 10.1016/j.ydbio.2013.11.015 PMC3971878

[b10] Liu W. . TRPM7 regulates gastrulation during vertebrate embryogenesis. Dev. Biol. 350, 348–357 (2011). 21145885 10.1016/j.ydbio.2010.11.034 PMC3292586

[b11] Overton J. D. . Hepatocystin is essential for TRPM7 function during early embryogenesis. Sci Rep. 5, 18395 (2015). 26671672 10.1038/srep18395 PMC4680877

[b12] Ryazanov A. G., Pavur K. S. & Dorovkov M. V. Alpha-kinases: a new class of protein kinases with a novel catalytic domain. Curr Biol. 9, R43–R45 (1999). 10.1016/s0960-9822(99)80006-2 10021370

[b13] Ryazanov A. G. . Identification of a new class of protein kinases represented by eukaryotic elongation factor-2 kinase. PNAS 94, 4884–4889 (1997). 9144159 10.1073/pnas.94.10.4884 PMC24600

[b14] Demeuse P., Penner R. & Fleig A. TRPM7 channel is regulated by magnesium nucleotides via its kinase domain. J Gen Physiol. 127, 421–434 (2006). 16533898 10.1085/jgp.200509410 PMC2151514

[b15] Yamaguchi H., Matsushita M., Nairn A. C. & Kuriyan J. Crystal structure of the atypical protein kinase domain of a TRP channel with phosphotransferase activity. Mol Cell. 7, 1047–1057 (2001). 11389851 10.1016/s1097-2765(01)00256-8

[b16] Drennan D. & Ryazanov A. G. Alpha-kinases: analysis of the family and comparison with conventional protein kinases. Prog Biophys Mol Biol. 85, 1–32 (2004). 15050379 10.1016/S0079-6107(03)00060-9

[b17] Crawley S. W. & Cote G. P. Identification of dimer interactions required for the catalytic activity of the TRPM7 alpha-kinase domain. Biochem J 420, 115–122 (2009). 19228120 10.1042/BJ20081405

[b18] van der Wijst J. . Kinase and channel activity of TRPM6 are co-ordinated by a dimerization motif and pocket interaction. Biochem J 460, 165–175 (2014). 24650431 10.1042/BJ20131639 PMC4019984

[b19] Clark K. . TRPM7 regulates myosin IIA filament stability and protein localization by heavy chain phosphorylation. J Mol Biol. 378, 790–803 (2008). 18394644 10.1016/j.jmb.2008.02.057 PMC4541798

[b20] Clark K. . The alpha-kinases TRPM6 and TRPM7, but not eEF-2 kinase, phosphorylate the assembly domain of myosin IIA, IIB and IIC. FEBS lett. 582, 2993–2997 (2008). 18675813 10.1016/j.febslet.2008.07.043

[b21] Dorovkov M. V. & Ryazanov A. G. Phosphorylation of annexin I by TRPM7 channel-kinase. J Biol Chem. 279, 50643–50646 (2004). 15485879 10.1074/jbc.C400441200

[b22] Deason-Towne F., Perraud A. L. & Schmitz C. Identification of Ser/Thr phosphorylation sites in the C2-domain of phospholipase C gamma2 (PLCgamma2) using TRPM7-kinase. Cell Signal. 24, 2070–2075 (2012). 22759789 10.1016/j.cellsig.2012.06.015 PMC4049354

[b23] Desai B. N. . Cleavage of TRPM7 releases the kinase domain from the ion channel and regulates its participation in Fas-induced apoptosis. Dev cell. 22, 1149–1162 (2012). 22698280 10.1016/j.devcel.2012.04.006 PMC3397829

[b24] Clark K. . Massive autophosphorylation of the Ser/Thr-rich domain controls protein kinase activity of TRPM6 and TRPM7. PloS one 3, e1876 (2008). 18365021 10.1371/journal.pone.0001876 PMC2267223

[b25] Kim T. Y., Shin S. K., Song M.-Y., Lee J. E. & Park K.-S. Identification of the phosphorylation sites on intact TRPM7 channels from mammalian cells. Biochem Biophys Res Commun. 417, 1030–1034 (2012). 22222377 10.1016/j.bbrc.2011.12.085

[b26] Moore C. E., Regufe da Mota S., Mikolajek H. & Proud C. G. A conserved loop in the catalytic domain of eukaryotic elongation factor 2 kinase plays a key role in its substrate specificity. Mol Cell Biol. 34, 2294–2307 (2014). 24732796 10.1128/MCB.00388-14 PMC4054288

[b27] Crawley S. W. . Autophosphorylation activates *Dictyostelium* myosin II heavy chain kinase A by providing a ligand for an allosteric binding site in the alpha-kinase domain. J Biol Chem. 286, 2607–2616 (2011). 21071445 10.1074/jbc.M110.177014 PMC3024756

[b28] Schmitz C. . The channel kinases TRPM6 and TRPM7 are functionally nonredundant. J Biol Chem. 280, 37763–37771 (2005). 16150690 10.1074/jbc.M509175200

[b29] Brandao K., Deason-Towne F., Zhao X., Perraud A. L. & Schmitz C. TRPM6 kinase activity regulates TRPM7 trafficking and inhibits cellular growth under hypomagnesic conditions. Cell Mol Life Sci. 71, 4853–4867 (2014). 24858416 10.1007/s00018-014-1647-7 PMC4234683

[b30] Li M., Jiang J. & Yue L. Functional characterization of homo- and heteromeric channel kinases TRPM6 and TRPM7. J Gen Physiol. 127, 525–537 (2006). 16636202 10.1085/jgp.200609502 PMC2151519

[b31] Nadler M. J. . LTRPC7 is a Mg.ATP-regulated divalent cation channel required for cell viability. Nature. 411, 590–595 (2001). 11385574 10.1038/35079092

[b32] Krapivinsky G., Krapivinsky L., Manasian Y. & Clapham D. E. The TRPM7 chanzyme is cleaved to release a chromatin-modifying kinase. Cell. 157, 1061–1072 (2014). 24855944 10.1016/j.cell.2014.03.046 PMC4156102

[b33] Fetrow J. S. Omega loops: nonregular secondary structures significant in protein function and stability. The FASEB J 9, 708–717 (1995). 7601335

[b34] Matsushita M. . Channel function is dissociated from the intrinsic kinase activity and autophosphorylation of TRPM7/ChaK1. J Biol Chem. 280, 20793–20803 (2005). 15781465 10.1074/jbc.M413671200

[b35] Amanchy R. . A curated compendium of phosphorylation motifs. Nat Biotech. 25, 285–286 (2007). 10.1038/nbt0307-285 17344875

[b36] Runnels L. W., Yue L. & Clapham D. E. TRP-PLIK, a bifunctional protein with kinase and ion channel activities. Science. 291, 1043–1047 (2001). 11161216 10.1126/science.1058519

[b37] Runnels L. W., Yue L. & Clapham D. E. The TRPM7 channel is inactivated by PIP(2) hydrolysis. Nat Cell Biol. 4, 329–336 (2002). 11941371 10.1038/ncb781

[b38] Ye Q., Crawley S. W., Yang Y., Cote G. P. & Jia Z. Crystal structure of the alpha-kinase domain of *Dictyostelium* myosin heavy chain kinase A. Sci Signal. 3, ra17 (2010). 20197546 10.1126/scisignal.2000525 PMC2894936

[b39] Su L. T. . TRPM7 activates m-calpain by stress-dependent stimulation of p38 MAPK and c-Jun N-terminal kinase. J Mol Biol. 396, 858–869 (2010). 20070945 10.1016/j.jmb.2010.01.014 PMC2825087

[b40] Li M. . Molecular determinants of Mg2+ and Ca2+ permeability and pH sensitivity in TRPM6 and TRPM7. J Biol Chem. 282, 25817–25830 (2007). 17599911 10.1074/jbc.M608972200 PMC3239414

[b41] Voets T. . TRPM6 forms the Mg2+ influx channel involved in intestinal and renal Mg2+ absorption. J Biol Chem 279, 19–25 (2004). 14576148 10.1074/jbc.M311201200

[b42] Huse M. & Kuriyan J. The conformational plasticity of protein kinases. Cell. 109, 275–282 (2002). 12015977 10.1016/s0092-8674(02)00741-9

[b43] Yang Y., Ye Q., Jia Z. & Cote G. P. Characterization of the catalytic and nucleotide binding properties of the alpha-kinase domain of *Dictyostelium* myosin-II heavy chain kinase A. J Biol Chem. 290, 23935–23946 (2015). 26260792 10.1074/jbc.M115.672410 PMC4583013

[b44] Tavares C. D. . The molecular mechanism of eukaryotic elongation factor 2 kinase activation. J Biol Chem. 289, 23901–23916 (2014). 25012662 10.1074/jbc.M114.577148 PMC4156036

[b45] Ye Q. . Structure of the *Dictyostelium* myosin-II heavy chain kinase A (MHCK-A) alpha-kinase domain apoenzyme reveals a novel autoinhibited conformation. Sci Rep. 6, 26634 (2016). 27211275 10.1038/srep26634 PMC4876393

