# Supplementary material for: Mass Spectrometric Analysis of TRPM6 and TRPM7 Phosphorylation Reveals Regulatory Mechanisms of the Channel-Kinases
Source: Sci Rep. 2017 Feb 21;7:42739. doi: 10.1038/srep42739 (PMC5318989; doi:10.1038/srep42739)
Supplement: Supplementary Information [file srep42739-s1.pdf]

Mass Spectrometric Analysis of TRPM6 and TRPM7 Phosphorylation Reveals Regulatory Mechanisms of the Channel-Kinases

Na Cai[1], Zhiyong Bai[1], Vikas Nanda[2] & Loren W. Runnels[1,*].

Rutgers-Robert Wood Johnson Medical School, [1]Dept. of Pharmacology and [2]Dept. of Biochemistry and Molecular Biology, Piscataway, 08854, U.S.A. *runnellw@rwjms.rutgers.edu

Supplementary Information

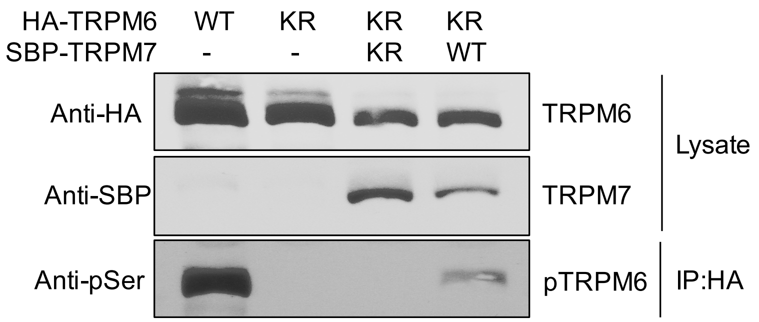

Supplementary Figure 1. TRPM7 transphosphorylates TRPM6 *in vivo*. HA-tagged human TRPM6 (WT) and TRPM6-K1804R (KR) were transiently expressed individually or co-expressed with SBP-tagged mouse TRPM7 (WT) and TRPM7-K1646R (KR) in HEK-293T cells. The proteins were immunopurified by HA-agarose. Proteins in the lysate and the immunoprecipitated samples were resolved by SDS-PAGE and analyzed by western blotting with the indicated antibodies.

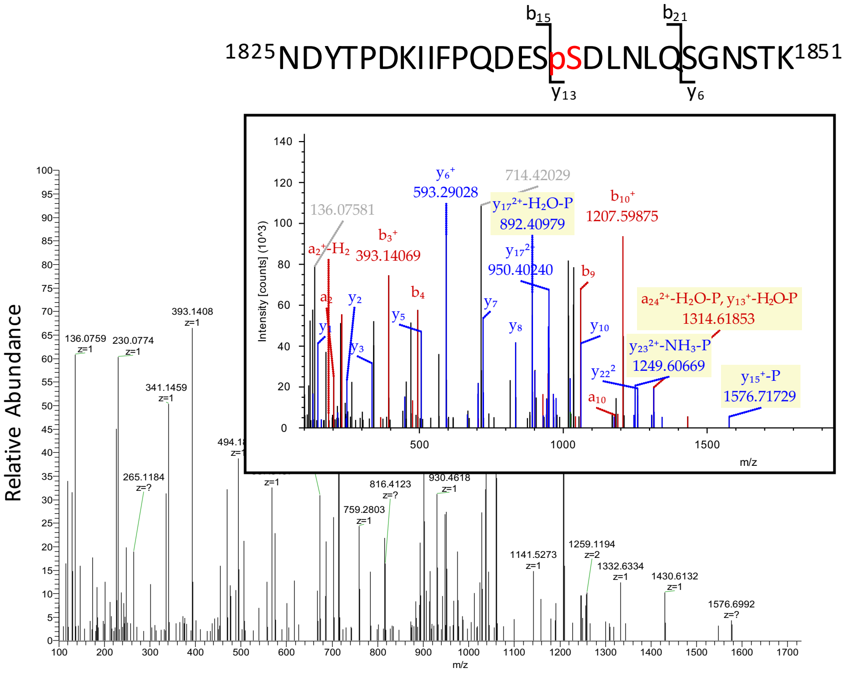

Supplementary Figure 2. Phosphorylation analysis of Sumo-TRPM7-Kinase. Representative MS/MS spectra of a peptide containing phosphorylation site from purified Sumo-TRPM7-Kinase WT. Phosphorylation on S1840 was identified on the tryptic phosphopeptide NDYTPDKIIFPQDESpSDLNLQSGNSTK (m/z observed at 1036.133 in +3 charge state).

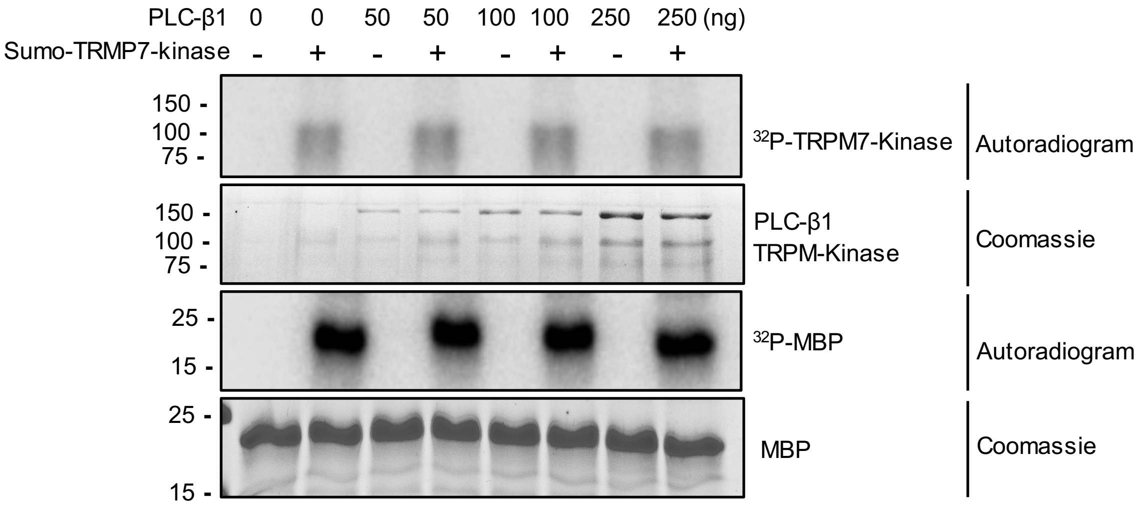

Supplementary Figure 3. PLC-β1 does not stimulate TRPM7 kinase activity. SBP-tagged PLC-β1 was expressed in a tetracycline-inducible HEK-293 cell line, immunoprecipitated by streptavidin agarose, and eluted by biotin. Increasing amounts of purified PLC-β1 was added to *in vitro* kinase reactions in the presence of Sumo-TRPM7-Kinase and MBP. The reactions were performed at 30°C for 5 min. The proteins were resolved by SDS-PAGE and Coomassie blue staining. Phosphorylation was detected by autoradiography.

Supplementary Table 1.

Phosphopeptides identified from tryptic digestions of HA-mTRPM7-WT and K1646R.

HA-mTRPM7 Phosphorylation				#PSMs	
Positions in Master Proteins	Sequence	Modifications	Phosphorylation in Master Proteins	WT	K1646R
Q923J1 [83-105]	HTEQSPTDAYGVINFQGGSHSYR	S101+Phospho;	S101		1
Q923J1 [547-560]	SGRNTSSSTPQLRK	N550+Deamidated; S547+Phospho;	S547	1	1
Q923J1 [1224-1247]	SLQSLDSQIGHLQDLSALTVDTLK	S1224+Phospho;	S1224	1	
Q923J1 [1248-1259]	TLTAQKASEASK	S1255+Phospho;	S1255	1	
Q923J1 [1248-1266]	TLTAQKASEASKVHNEITR	S1255+Phospho;	S1255	2	
Q923J1 [1254-1266]	ASEASKVHNEITR	S1258+Phospho;	S1258	1	
Q923J1 [1267-1289]	ELSISKHLAQNLIDDVPVRPLWK	S1269+Phospho;	S1269	3	1
Q923J1 [1267-1289]	ELSISKHLAQNLIDDVPVRPLWK	S1271+Phospho;	S1271	1	
Q923J1 [1290-1306]	KPSAVNTLSSSLPQGDR	S1298+Phospho;	S1298	3	
Q923J1 [1340-1365]	KEFNIPEAGSSCGALFPSAVSPPELR	C1351+Carbamidomethyl; S1360+Phospho;	S1360	1	
Q923J1 [1341-1365]	EFNIPEAGSSCGALFPSAVSPPELR	C1351+Carbamidomethyl; S1360+Phospho;	S1360	3	2
Q923J1 [1350-1365]	SCGALFPSAVSPPELR	C1351+Carbamidomethyl; S1350+Phospho;	S1350	1	
Q923J1 [1351-1365]	CGALFPSAVSPPELR	C1351+Carbamidomethyl; S1357+Phospho;	S1357	4	1
Q923J1 [1383-1399]	LGSSPNSSPHMSSPPTK	M1393+Oxidation; S1395+Phospho;	S1395	1	
Q923J1 [1383-1399]	LGSSPNSSPHMSSPPTK	S1386+Phospho;	S1386	1	1
Q923J1 [1485-1498]	TSTSLHSVQESCSR	C1496+Carbamidomethyl; T1485+Phospho;	T1485	1	
Q923J1 [1500-1512]	RASTEDSPEVDSK	S1502+Phospho;	S1502	1	1
Q923J1 [1500-1521]	RASTEDSPEVDSKAALLPDWLR	S1511+Phospho;	S1511	1	
Q923J1 [1528-1558]	EMPSEGGTLNGLASPFKPVLDTNYYYSAVER	M1529+Oxidation; S1541+Phospho;	S1541	1	1
Q923J1 [1564-1576]	LSQSIPFVPVPPR	S1565+Phospho;	S1565	3	
Q923J1 [1564-1576]	LSQSIPFVPVPPR	S1567+Phospho;	S1567	27	
Q923J1 [1564-1584]	LSQSIPFVPVPPRGEPVTVYR	S1567+Phospho;	S1567	6	
Q923J1 [1693-1699]	SIPYSPR	S1693+Phospho;	S1693	1	
Q923J1 [1825-1851]	NDYTPDKIIFPQDESSDLNLQSGNSTK	S1839+Phospho;	S1839	1	
Q923J1 [1825-1851]	NDYTPDKIIFPQDESSDLNLQSGNSTK	T1828+Phospho;	T1828	1	1
Q923J1 [1825-1860]	NDYTPDKIIFPQDESSDLNLQSGNSTKESEATNSVR	S1846+Phospho;	S1846	1	
Q923J1 [1825-1860]	IIFPQDESSDLNLQSGNSTKESEATNSVR	S1849+Phospho;	S1849	1	

Supplementary Table 2.

Phosphopeptides identified from tryptic digestions of FLAG-mTRPM7.

FLAG-mTRPM7 Phosphorylation				
Positions in Master Proteins	Sequence	Modifications	Phosphorylation in Master Proteins	# PSMs
Q923J1 [83-105]	HTEQSPTDAYGVINFQGGSHSYR	S101+Phospho	S101	4
Q923J1 [820-835]	ILDSSDGKNEMEIHIK	S823+Phospho;	S823	1
Q923J1 [1224-1247]	SLQSLDSQIGHLQDLSALTVDTLK	Q1236+Deamidated; S1227+Phospho;	S1227	1
Q923J1 [1224-1247]	SLQSLDSQIGHLQDLSALTVDTLK	S1230+Phospho;	S1230	1
Q96QT4 [1248-1259]	TLTAQKASEASK	S1255+Phospho	S1255	1
Q923J1 [1248-1266]	TLTAQKASEASKVHNEITR	S1255+Phospho;	S1255	1
Q923J1 [1254-1266]	ASEASKVHNEITR	S1255+Phospho; S1258+Phospho;	S1255/S1258	1
Q923J1 [1267-1289]	ELSISKHLAQNLIDDVPVRPLWK	S1269+Phospho;	S1269	1
Q923J1 [1341-1365]	EFNIPEAGSSCGALFPSAVSPPELR	C1351+Carbamidomethyl; S1360+Phospho;	S1360	11
Q923J1 [1383-1399]	LGSSPNSSPHMSSPPTK	S1386+Phospho;	S1386	1
Q923J1 [1383-1399]	LGSSPNSSPHMSSPPTK	S1389+Phospho;	S1389	2
Q923J1 [1383-1399]	LGSSPNSSPHMSSPPTK	S1394+Phospho;	S1394	1
Q923J1 [1383-1399]	LGSSPNSSPHMSSPPTK	S1395+Phospho;	S1395	2
Q923J1 [1383-1399]	LGSSPNSSPHMSSPPTK	M1393+Oxidation; S1386+Phospho;	S1386	2
Q923J1 [1383-1399]	LGSSPNSSPHMSSPPTK	M1393+Oxidation; S1389+Phospho;	S1389	1
Q923J1 [1383-1399]	LGSSPNSSPHMSSPPTK	M1393+Oxidation; S1394+Phospho;	S1394	1
Q923J1 [1383-1399]	LGSSPNSSPHMSSPPTK	M1393+Oxidation; S1395+Phospho;	S1395	1
Q923J1 [1400-1411]	FSVSTPSQPSCK	C1410+Carbamidomethyl; S1403+Phospho;	S1403	1
Q923J1 [1500-1512]	RASTEDSPEVDSK	S1502+Phospho;	S1502	2
Q923J1 [1564-1576]	LSQSIPFVPVPPR	S1565+Phospho;	S1565	1
Q923J1 [1564-1576]	LSQSIPFVPVPPR	S1567+Phospho;	S1567	5
Q923J1 [1564-1584]	LSQSIPFVPVPPRGEPVTVYR	S1567+Phospho;	S1567	1
Q923J1 [1609-1622]	IEFLSKEEMGGGLR	S1613+Phospho;	S1613	1
Q923J1 [1824-1851]	RNDYTPDKIIFPQDESSDLNLQSGNSTK	T1828+Phospho;	T1828	1
Q923J1 [1825-1860]	IIFPQDESSDLNLQSGNSTKESEATNSVR	S1839+Phospho;	S1839	2
Q923J1 [1825-1860]	NDYTPDKIIFPQDESSDLNLQSGNSTKESEATNSVR	Q1836+Deamidated; S1839+Phospho;	S1839	1
Q923J1 [1825-1860]	IIFPQDESSDLNLQSGNSTKESEATNSVR	S1839+Phospho; S1840+Phospho;	S1839/S1840	1
Q923J1 [1825-1860]	IIFPQDESSDLNLQSGNSTKESEATNSVR	S1849+Phospho;	S1849	1

Supplementary Table 3.

Phosphopeptides identified from tryptic digestions of GFP-mTRPM7-Cterm-WT and K1646R.

GFP-mTRPM7-Cterm Phosphorylation				#PSMs	
Positions in Master Proteins	Sequence	Modifications	Phosphorylation in Master Proteins	WT	K1646R
Q923J1 [1224-1147]	SLQSLDSQIGHLQDLSALTVDTLK	S1230+Phospho;	S1230	1	
Q923J1 [1290-1306]	KPSAVNTLSSSLPQGDR	S1299+Phospho;	S1299	1	
Q923J1 [1341-1365]	EFNIPEAGSSCGALFPSAVSPPELR	C1351+Carbamidomethyl; S1360+Phospho;	S1360	1	
Q923J1 [1383-1399]	LGSSPNSSPHMSSPPTK	M1393+Oxidation; S1386+Phospho;	S1386	2	
Q923J1 [1383-1399]	LGSSPNSSPHMSSPPTK	M1393+Oxidation; S1389+Phospho;	S1389	1	
Q923J1 [1383-1399]	LGSSPNSSPHMSSPPTK	M1393+Oxidation; S1390+Phospho;	S1390		1
Q923J1 [1383-1399]	LGSSPNSSPHMSSPPTK	M1393+Oxidation; S1395+Phospho;	S1395		1
Q923J1 [1383-1399]	LGSSPNSSPHMSSPPTK	S1389+Phospho;	S1389	4	1
Q923J1 [1383-1399]	LGSSPNSSPHMSSPPTK	S1394+Phospho;	S1394		2
Q923J1 [1400-1411]	FSVSTPSQPSCK	C1410+Carbamidomethyl; S1403+Phospho;	S1403	2	
Q923J1 [1500-1512]	RASTEDSPEVDSK	S1502+Phospho;	S1502	5	
Q923J1 [1500-1521]	ASTEDSPEVDSKAALLPDWLR	S1506+Phospho;	S1506	1	
Q923J1 [1564-1576]	LSQSIPFVPVPPR	S1567+Phospho;	S1567	10	

Supplementary Table 4.

Phosphopeptides identified from tryptic digestions of SBP-mTRPM7-K1646R.

Cross-phosphorylation of mTRPM7 by hTRPM6			#PSMs	
Positions in Master Proteins	Sequence	Phosphorylation in Master Proteins	Co-expression with HA-hTRPM6	Expressed alone
Q923J1 [83-105]	HTEQSPTDAYGVINFQGGSHSYR	S87+Phospho;	4	4
Q923J1 [83-105]	HTEQSPTDAYGVINFQGGSHSYR	T89+Phospho;	1	
Q923J1 [83-105]	HTEQSPTDAYGVINFQGGSHSYR	S101+Phospho;	7	5
Q923J1 [331-351]	QTEEGGNLPDAAEPDIISTIK	T332+Phospho;	1	1
Q923J1 [550-559]	NTSSSTPQLR	T551+Phospho;	1	
Q923J1 [550-559]	NTSSSTPQLR	S552+Phospho;	2	3
Q923J1 [550-559]	NTSSSTPQLR	S553+Phospho;		1
Q923J1 [550-559]	NTSSSTPQLR	S554+Phospho;	2	
Q923J1 [550-560]	NTSSSTPQLRK	S552+Phospho;	2	4
Q923J1 [550-560]	NTSSSTPQLRK	S554+Phospho;	1	1
Q923J1 [550-568]	NTSSSTPQLRKSHETFGNR	S561+Phospho;	2	2
Q923J1 [780-816]	AEMSHIPQSQDAHQMTMEDSENNFHNITEEIPMEVFK	S788+Phospho;		1
Q923J1 [1248-1259]	TLTAQKASEASK	S1255+Phospho;	3	
Q923J1 [1248-1266]	TLTAQKASEASKVHNEITR	T1250+Phospho;	2	
Q923J1 [1248-1266]	TLTAQKASEASKVHNEITR	S1255+Phospho;	10	
Q923J1 [1290-1306]	KPSAVNTLSSSLPQGDR	S1292+Phospho;	1	2
Q923J1 [1290-1306]	KPSAVNTLSSSLPQGDR	S1299+Phospho;	2	
Q923J1 [1290-1306]	KPSAVNTLSSSLPQGDR	S1300+Phospho;	2	
Q923J1 [1341-1365]	EFNIPEAGSSCGALFPSAVSPPELR	S1349+Phospho;	1	
Q923J1 [1340-1365]	KEFNIPEAGSSCGALFPSAVSPPELR	S1360+Phospho;	2	1
Q923J1 [1341-1365]	EFNIPEAGSSCGALFPSAVSPPELR	S1360+Phospho;	7	6
Q923J1 [1383-1399]	LGSSPNSSPHMSSPPTK	S1386+Phospho;		4
Q923J1 [1383-1399]	LGSSPNSSPHMSSPPTK	S1390+Phospho;	1	
Q923J1 [1383-1399]	LGSSPNSSPHMSSPPTK	S1395+Phospho;	9	4
Q923J1 [1383-1411]	LGSSPNSSPHMSSPPTKFSVSTPSQPSCK	S1386+Phospho;	4	4
Q923J1 [1383-1411]	LGSSPNSSPHMSSPPTKFSVSTPSQPSCK	S1390+Phospho;	1	
Q923J1 [1383-1411]	LGSSPNSSPHMSSPPTKFSVSTPSQPSCK	S1395+Phospho;	5	5
Q923J1 [1400-1411]	FSVSTPSQPSCK	S1403+Phospho;	3	
Q923J1 [1412-1427]	SHLESTTKDQEPIFYK	S1416+Phospho;	2	
Q923J1 [1428-1450]	AAEGDNIEFGAFVGHRDSMDLQR	S1445+Phospho;	5	1
Q923J1 [1460-1472]	ELLSNDTPENTLK	T1466+Phospho;	2	
Q923J1 [1485-1498]	TSTSLHSVQESCSR	T1487+Phospho;	4	
Q923J1 [1485-1498]	TSTSLHSVQESCSR	S1495+Phospho;		1
Q923J1 [1485-1512]	TSTSLHSVQESCSRRASTEDSPEVDSK	S1491+Phospho; S1495+Phospho;	1	
Q923J1 [1485-1512]	TSTSLHSVQESCSRRASTEDSPEVDSK	S1495+Phospho; S1497+Phospho;	1	2
Q923J1 [1500-1512]	RASTEDSPEVDSK	S1502+Phospho;	7	9
Q923J1 [1500-1512]	RASTEDSPEVDSK	S1506+Phospho;	4	2
Q923J1 [1501-1512]	ASTEDSPEVDSK	S1506+Phospho;	1	2
Q923J1 [1501-1521]	ASTEDSPEVDSKAALLPDWLR	S1502+Phospho; S1506+Phospho;	1	
Q923J1 [1500-1521]	RASTEDSPEVDSKAALLPDWLR	S1502+Phospho; S1506+Phospho; T1503+Phospho;	2	
Q923J1 [1500-1521]	RASTEDSPEVDSKAALLPDWLR	S1502+Phospho; S1511+Phospho;	2	
Q923J1 [1501-1521]	ASTEDSPEVDSKAALLPDWLR	S1506+Phospho;	2	1
Q923J1 [1500-1521]	RASTEDSPEVDSKAALLPDWLR	S1506+Phospho; S1511+Phospho;	1	
Q923J1 [1501-1521]	ASTEDSPEVDSKAALLPDWLR	S1511+Phospho;		1
Q923J1 [1528-1558]	EMPSEGGTLNGLASPFKPVLDTNYYYSAVER	S1541+Phospho;	3	3
Q923J1 [1564-1576]	LSQSIPFVPVPPR	S1567+Phospho;	15	
Q923J1 [1564-1584]	LSQSIPFVPVPPRGEPVTVYR	S1565+Phospho;	3	
Q923J1 [1564-1584]	LSQSIPFVPVPPRGEPVTVYR	S1567+Phospho;	4	1
Q923J1 [1693-1699]	SIPYSPR	S1693+Phospho;	1	
Q923J1 [1825-1851]	NDYTPDKIIFPQDESSDLNLQSGNSTK	S1839+Phospho;		1
Q923J1 [1824-1851]	RNDYTPDKIIFPQDESSDLNLQSGNSTK	T1828+Phospho;	6	6
Q923J1 [1825-1851]	NDYTPDKIIFPQDESSDLNLQSGNSTK	T1828+Phospho;	3	5

Supplementary Table 5.
Phosphopeptides identified from tryptic digestions of HA-hTRPM6.

HA-hTRPM6 Phosphorylation Positions in Master Proteins	Sequence	Phosphorylation in Master Proteins	# PSMs
Q9BX84[10-18]	LQSQKSWIK	S12+Phospho;	3
Q9BX84[10-18]	LQSQKSWIK	S15+Phospho;	1
Q9BX84[25-38]	ECSTIIPSSKNPHR	S32+Phospho;	2
Q9BX84[86-110]	HTTKSPTDTFGTINFQDGEHTHHAK	S90+Phospho;	1
Q9BX84[86-110]	HTTKSPTDTFGTINFQDGEHTHHAK	S90+Phospho;	4
Q9BX84[116-185]	AAETTGAWIITEGINTGVSK	T170+Phospho;	3
Q9BX84[201-217]	KIWTVGIPPWGVIENQR	T204+Phospho;	2
Q9BX84[202-217]	IWTVGIPPWGVIENQR	T204+Phospho;	1
Q9BX84[238-258]	LTTLNSMHSHFILSDDGTVGK	T239+Phospho;	4
Q9BX84[238-258]	LTTLNSMHSHFILSDDGTVGK	T240+Phospho;	2
Q9BX84[238-258]	LTTLNSMHSHFILSDDGTVGK	S243+Phospho;	2
Q9BX84[547-569]	HQRHSSGNRNESAESTLHSQFIR	S551+Phospho;	1
Q9BX84[547-569]	HQRHSSGNRNESAESTLHSQFIR	S551+Phospho; S552+Phospho;	1
Q9BX84[550-569]	HSSGNRNESAESTLHSQFIR	S551+Phospho; S552+Phospho;	2
Q9BX84[547-569]	HQRHSSGNRNESAESTLHSQFIR	S551+Phospho; S558+Phospho;	1
Q9BX84[550-569]	HSSGNRNESAESTLHSQFIR	S552+Phospho;	48
Q9BX84[550-569]	HSSGNRNESAESTLHSQFIR	S558+Phospho;	4
Q9BX84[556-569]	NESAESTLHSQFIR	S558+Phospho;	3
Q9BX84[550-569]	HSSGNRNESAESTLHSQFIR	S561+Phospho;	2
Q9BX84[576-585]	FKEKSIVLHK	S580+Phospho;	1
Q9BX84[580-587]	SIVLHKSR	S586+Phospho;	1
Q9BX84[588-619]	KKSKEQNVSDDPESTGFLYPYNDLLVWAVLMK	S596+Phospho;	1
Q9BX84[686-700]	QNERMAMTLLTYELR	T693+Phospho;	2
Q9BX84[766-792]	AEMSHVPQSQDFQFMWYYSDQNASSSK	S769+Phospho;	3
Q9BX84[793-804]	ESASVKEYDLER	S794+Phospho;	4
Q9BX84[793-804]	ESASVKEYDLER	S796+Phospho;	6
Q9BX84[805-829]	GHDEKLDENQHFGLESGHQHLPWTR	S820+Phospho;	1
Q9BX84[997-1011]	AILSPKEPPSWSLAR	S1000+Phospho;	5
Q9BX84[1199-1223]	DSLLSLDSQVGHLQDLSALTVDTLK	S1203+Phospho;	1
Q9BX84[1224-1240]	VLSAVDTLQEDEALLAK	S1226+Phospho;	3
Q9BX84[1224-1240]	VLSAVDTLQEDEALLAK	T1230+Phospho;	1
Q9BX84[1272-1284]	KYQYYSMPSSLLR	S1277+Phospho;	6
Q9BX84[1273-1284]	YQYYSMPSSLLR	S1277+Phospho;	6
Q9BX84[1273-1284]	YQYYSMPSSLLR	S1277+Phospho; S1280+Phospho;	1
Q9BX84[1272-1284]	KYQYYSMPSSLLR	S1280+Phospho;	1
Q9BX84[1273-1284]	YQYYSMPSSLLR	S1280+Phospho;	3
Q9BX84[1285-1294]	SLAGGRHPPR	S1285+Phospho;	3
Q9BX84[1315-1335]	NDQERQETQSSIVVSGVSPNR	S1324+Phospho;	1
Q9BX84[1308-1335]	REATNVRNDQERQETQSSIVVSGVSPNR	S1324+Phospho; T1311+Phospho; T1322+Phospho;	1
Q9BX84[1315-1335]	NDQERQETQSSIVVSGVSPNR	S1325+Phospho;	1
Q9BX84[1315-1335]	NDQERQETQSSIVVSGVSPNR	S1332+Phospho;	6
Q9BX84[1315-1340]	NDQERQETQSSIVVSGVSPNRQAHSK	S1332+Phospho;	2
Q9BX84[1320-1335]	QETQSSIVVSGVSPNR	S1332+Phospho;	10
Q9BX84[1320-1340]	QETQSSIVVSGVSPNRQAHSK	S1332+Phospho;	1
Q9BX84[1341-1352]	YGQFLLVPSNLK	S1349+Phospho;	2
Q9BX84[1341-1353]	YGQFLLVPSNLKR	S1349+Phospho;	1
Q9BX84[1445-1470]	IMQTGGGYVNWAFSEGDETGVFSIKK	T1448+Phospho;	2
Q9BX84[1506-1531]	SAQSSECSEVGPWLQPNTSFWINPLR	S1506+Phospho;	1
Q9BX84[1506-1532]	SAQSSECSEVGPWLQPNTSFWINPLRR	S1506+Phospho;	1
Q9BX84[1506-1531]	SAQSSECSEVGPWLQPNTSFWINPLR	S1509+Phospho;	1
Q9BX84[1506-1532]	SAQSSECSEVGPWLQPNTSFWINPLRR	S1509+Phospho;	1
Q9BX84[1506-1532]	SAQSSECSEVGPWLQPNTSFWINPLRR	S1510+Phospho;	1
Q9BX84[1556-1572]	IKNLSGSSEIGQGAWVK	S1560+Phospho;	3
Q9BX84[1558-1572]	NLSGSSEIGQGAWVK	S1560+Phospho;	1
Q9BX84[1556-1572]	IKNLSGSSEIGQGAWVK	S1562+Phospho;	1
Q9BX84[1558-1572]	NLSGSSEIGQGAWVK	S1562+Phospho;	1
Q9BX84[1647-1662]	TKEIGQCAIQISDYLK	S1658+Phospho;	1
Q9BX84[1647-1670]	TKEIGQCAIQISDYLKQSQEDLSK	S1658+Phospho;	2
Q9BX84[1647-1670]	TKEIGQCAIQISDYLKQSQEDLSK	S1664+Phospho;	2
Q9BX84[1649-1670]	EIGQCAIQISDYLKQSQEDLSK	S1664+Phospho;	3
Q9BX84[1684-1695]	NSLLKSSIGVDK	S1689+Phospho;	1
Q9BX84[1689-1701]	SSIGVDKISASLK	S1697+Phospho;	1
Q9BX84[1689-1715]	SSIGVDKISASLKSPQEPHHHYSAIER	S1697+Phospho;	1
Q9BX84[1696-1715]	ISASLKSPQEPHHHYSAIER	S1699+Phospho;	3
Q9BX84[1696-1715]	ISASLKSPQEPHHHYSAIER	S1702+Phospho;	3
Q9BX84[1721-1742]	LSQTIPFTPVQLFAGEEITVYR	T1724+Phospho;	3
Q9BX84[1721-1753]	LSQTIPFTPVQLFAGEEITVYRLEESSPLNLDK	T1724+Phospho;	1
Q9BX84[1785-1804]	VVSTWSEDDILKPGQVFIVK	T1788+Phospho;	1
Q9BX84[1785-1804]	VVSTWSEDDILKPGQVFIVK	S1790+Phospho;	1
Q9BX84[1983-1999]	NDYSPERINSTFGLEIK	S1986+Phospho;	5
Q9BX84[2010-2022]	ETGRNSPEDDMQL	S2015+Phospho;	7

Supplementary Table 6.

Phosphopeptides identified from tryptic digestions of Sumo-mTRPM7-Kinase-WT and K1646R.

Sumo-mTRPM7-Kinase Phosphorylation				#PSMs	
Positions in Master Proteins	Sequence	Modifications	Phosphorylation in Master Proteins	WT	K1646R
Q923J1 [1400-1411]	FSVSTPSQPSCK	1xCarbamidomethyl; 1xPhospho	S1403	1	
Q923J1 [1400-1411]	FSVSTPSQPSCK	1xCarbamidomethyl; 1xPhospho	S1403	1	
Q923J1 [1412-1427]	SHLESTTKDQEPIFYK	1xPhospho	S1416/S1417/T1418	8	
Q923J1 [1428-1450]	AAEGDNIEFGAFVGHRDSMDLQR	1xPhospho	S1445	8	
Q923J1 [1428-1450]	AAEGDNIEFGAFVGHRDSMDLQR	1xOxidation; 1xPhospho	S1445	6	
Q923J1 [1428-1450]	AAEGDNIEFGAFVGHRDSMDLQR	1xPhospho	S1445	2	2
Q923J1 [1428-1450]	AAEGDNIEFGAFVGHRDSMDLQR	1xOxidation; 1xPhospho	S1445	2	2
Q923J1 [1458-1472]	IRELLSNDTPENTLK	1xPhospho	T1470	4	
Q923J1 [1458-1472]	IRELLSNDTPENTLK	1xPhospho	T1470	1	
Q923J1 [1460-1472]	ELLSNDTPENTLK	2xPhospho	S1463; T1470	2	
Q923J1 [1460-1472]	ELLSNDTPENTLK	1xPhospho	T1470	8	
Q923J1 [1460-1472]	ELLSNDTPENTLK	1xPhospho	T1470	3	
Q923J1 [1473-1484]	HVGAAGYSECCK	2xCarbamidomethyl; 1xPhospho	S1480	1	
Q923J1 [1500-1512]	RASTEDSPEVDSK	1xPhospho	S1511	3	
Q923J1 [1500-1521]	RASTEDSPEVDSKAALLPDWLR	1xPhospho	S1511	2	
Q923J1 [1500-1521]	RASTEDSPEVDSKAALLPDWLR	1xPhospho	S1511	1	
Q923J1 [1501-1512]	ASTEDSPEVDSK	1xPhospho	S1511	2	
Q923J1 [1501-1512]	ASTEDSPEVDSK	1xPhospho	S1511	1	
Q923J1 [1501-1521]	ASTEDSPEVDSKAALLPDWLR	1xPhospho	S1511	4	
Q923J1 [1501-1521]	ASTEDSPEVDSKAALLPDWLR	1xPhospho	S1511	5	
Q923J1 [1522-1558]	DRPSNREMPSEGGTLNGLASPFKPVLDTNYYYSAVER	1xOxidation; 1xPhospho	S1525	1	
Q923J1 [1528-1558]	EMPSEGGTLNGLASPFKPVLDTNYYYSAVER	1xOxidation; 1xPhospho	S1541	8	
Q923J1 [1528-1558]	EMPSEGGTLNGLASPFKPVLDTNYYYSAVER	1xOxidation; 1xPhospho	T1535	1	
Q923J1 [1564-1576]	LSQSIPFVPVPPR	1xPhospho	S1565	19	
Q923J1 [1564-1576]	LSQSIPFVPVPPR	1xPhospho	S1567	8	
Q923J1 [1564-1584]	LSQSIPFVPVPPRGEPVTVYR	1xPhospho	S1567	5	
Q923J1 [1564-1584]	LSQSIPFVPVPPRGEPVTVYR	1xPhospho	S1567	14	
Q923J1 [1647-1671]	SFLPEVINTWSSIYKEDTVLHLCLR	1xCarbamidomethyl; 1xPhospho	S1658	3	
Q923J1 [1647-1671]	SFLPEVINTWSSIYKEDTVLHLCLR	1xCarbamidomethyl; 1xPhospho	S1658	1	
Q923J1 [1678-1690]	AAQKLTFAFNQMK	1xOxidation; 1xPhospho	T1683	1	
Q923J1 [1678-1692]	AAQKLTFAFNQMKPK	1xOxidation; 1xPhospho	T1683	2	
Q923J1 [1678-1692]	AAQKLTFAFNQMKPK	1xPhospho	T1683	1	
Q923J1 [1678-1692]	AAQKLTFAFNQMKPK	1xOxidation; 1xPhospho	T1683	3	
Q923J1 [1682-1692]	LTFAFNQMKPK	1xPhospho	T1683	1	
Q923J1 [1682-1692]	LTFAFNQMKPK	1xOxidation; 1xPhospho	T1683	2	
Q923J1 [1682-1699]	LTFAFNQMKPKSIPYSPR	1xOxidation; 1xPhospho	S1693	2	
Q923J1 [1728-1758]	YNNNNGDEIIPTNTLEEIMLAFSHWTYEYTR	1xPhospho	T1741	3	
Q923J1 [1759-1780]	GELLVLDLQGVGENLTDPSVIK	1xPhospho	S1777	5	
Q923J1 [1759-1784]	GELLVLDLQGVGENLTDPSVIKAEEK	1xPhospho	S1777	2	
Q923J1 [1759-1784]	GELLVLDLQGVGENLTDPSVIKAEEK	1xPhospho	S1777	1	
Q923J1 [1825-1851]	NDYTPDKIIFPQDESSDLNLQSGNSTK	1xPhospho	S1840	8	
Q923J1 [1825-1851]	NDYTPDKIIFPQDESSDLNLQSGNSTK	1xPhospho	S1840	1	
Q923J1 [1832-1851]	IIFPQDESSDLNLQSGNSTK	1xPhospho	S1846	2	
Q923J1 [1852-1860]	ESEATNSVR	1xPhospho	S1858	1	
Q923J1 [1852-1863]	ESEATNSVRLML	1xOxidation; 1xPhospho	S1858	1	
Q923J1 [1852-1863]	ESEATNSVRLML	1xOxidation; 1xPhospho	S1858	3	
